# Novel insights into the rhizosphere and seawater microbiome of *Zostera marina* in diverse mariculture zones

**DOI:** 10.1186/s40168-024-01759-3

**Published:** 2024-02-14

**Authors:** Hao Sun, Tianyu Wang, Shuai Liu, Xiaoyu Tang, Jie Sun, Xuerui Liu, Ye Zhao, Pingping Shen, Yanying Zhang

**Affiliations:** 1 School of Ocean, Yantai University, Yantai, 264005 China; 2 CAS Key Laboratory of Tropical Marine Bio-resources and Ecology, South China Sea Institute of Oceanology, Guangzhou, 510301 China; 3 Fisheries College, Ocean University of China, Qingdao, 266003 China

**Keywords:** *Zostera marina*, Bacterial community, Eukaryotic community, Fungal community, Rhizosphere

## Abstract

**Background:**

Seagrasses offer various ecosystem services and possess high levels of primary productivity. However, the development of mariculture has affected the homeostasis of seagrass meadow ecosystems. Plant-microbiome associations are essential for seagrasses health, but little is known about the role of environmental microbiomes and how they affect seagrass in a mariculture environment. In this study, we investigated the influence of mariculture on the rhizosphere and seawater microbiome surrounding *Zostera marina* and focused on the bacterial, eukaryotic, and fungal components in the composition, diversity, metabolism, and responses to mariculture-related environmental factors.

**Results:**

Significant differences in the composition, richness, diversity, and internal relations of the bacterial community between the seawater and rhizosphere sediment surrounding *Z. marina* were observed, while differences in the eukaryotic and fungal communities were less significant. More complex bacterial and fungal co-occurrence networks were found in the seawater and rhizosphere sediment of the *Saccharina japonica* (SJ) and sea cucumber (SC) culture zones. The seawater in the SJ zone had higher levels of dissimilatory and assimilatory nitrate reduction, denitrification, and nitrogen fixation processes than the other three zones. The assimilatory sulfate reduction enzymes were higher in the rhizosphere sediments of the SJ zone than in the other three zones. Tetracycline, sulfonamide, and diaminopyrimidine resistance genes were enriched in the mariculture SJ and SC zones.

**Conclusions:**

Our findings might contribute to a better understanding of the effects of mariculture on the seagrass and the meadow ecosystems and thus revealing their potential operating mechanisms. These insights may serve to raise awareness of the effects of human activities on natural ecosystems, regulation of antibiotic usage, and environmental restoration.

Video Abstract

**Supplementary Information:**

The online version contains supplementary material available at 10.1186/s40168-024-01759-3.

## Background

Seagrasses are found along coastlines worldwide and are the only flowering plants that have adapted to the underwater environment [1]. Seagrass meadows play a vital role in coastal ecosystems. They act as carbon sinks, burying up to 27.4 Tg of marine organic carbon annually [2]. One widespread species, *Zostera marina* (eelgrass), provides living habitat and food for marine organisms along coasts throughout the Northern Hemisphere [3, 4]. Recent studies have revealed that plant-microbe associations, which facilitate several metabolic exchanges and biogeochemical transformations thus altering the homeostasis of their physiological and biological functions in seagrass meadow ecosystems, are essential for seagrass health [4–7]. It has been reported that the presence of microbial community is crucial for developing seagrasses, including seed germination, phytohormone production, and the formation of defense mechanisms against pathogens [8, 9].

The rhizosphere has a rich diversity of microorganisms, which are beneficial to plants through the actions of pathogenic infection suppressions and help in acquiring nutrients [10]. Seagrass rhizosphere sediments and surrounding seawaters are hotspots of nitrogen and sulfur cycles, processes that are governed by specific microorganisms, particularly bacteria [11, 12]. These processes include nitrogen fixation and nitrification [13], ammonium production [14], sulfide oxidation, and sulfate reduction [4, 13]. Research has demonstrated that fungi are crucial for the fitness and health of terrestrial plants and act as pathogens or endophytes, but the role of fungi in marine habitats has been poorly studied [15]. It is thought fungi potentially play important roles in the cycle of organic matter and the dynamics of food webs in marine ecosystems, such as seagrass meadows [16, 17]. Recently, studies have investigated the diversity of the seagrass-associated fungal community and emphasized the importance of understanding the variables that influence the dynamics of these communities [18, 19]. Seagrass meadows commonly co-exist with algae, especially in tropical oceans [20], in which affect the stability and primary productivity of the seagrass community, and ultimately affect their carbon sink [21, 22]. Previous studies have shown that the red *Smithora* algae persists with the seagrass *Z. marina* and influences the seagrass-associated assemblages [23]. Seagrass-associated microorganisms, including bacteria, fungi, and eukaryotic algae, have increasingly been recognized as key components of seagrass ecology [8, 24].

Maricultures, constructed on coastal lines, are crucial for supplying marine seafood for humans, raising the incomes of coastal communities, and promoting the development of a marine economy. However, it will inevitably impact the original ecological homeostasis [25]. China has the largest mariculture area and production in Asia, which accounts for ~71% of the global mariculture production [26]. Over the past few decades, mariculture has grown at an annual rate of ~6% in China [27]. The rapid development of mariculture has unfortunately resulted in environmental issues, such as marine pollution, eutrophication, antibiotic residues, and food safety concerns [28]. The residues of antibiotics enrich the antibiotic resistance genes (ARGs) in the water columns and sediments, change the microbial communities and biogeochemical cycles, and eventually threaten the lives of marine organisms and human health [29, 30]. Some bacterial taxa, such as *Vibrio* and *Mycobacterium*, are strongly linked to the ARGs and contribute to the ARG enrichment [31, 32].

The microbiome in seawater and rhizosphere sediment of seagrass may change when exposed to different interfering factors. Sea cucumbers are associated with seagrass meadows in the natural marine environment and form complex interrelationships with the seagrass [33]. Seagrasses could protect sea cucumbers from predators, while sea cucumbers, in turn, may act as bioturbators or ecosystem engineers to promote the transformation of nutrients in the rhizospheres of seagrasses [34]. However, sea cucumbers mariculture may alter the microbiomes and metabolic processes of natural seagrass meadows [33]. Active seagrass restoration is an important tool for facilitating seagrass meadow recovery, as such restoration produces fewer physical stressors and provides more positive intraspecific interactions that may enhance seagrass growth [35]. Positive interactions, such as mutualistic effects, are common in natural seagrass meadows [36], but those in the seagrass restoration areas may be different. Some studies reported that kelp cultivation did not impact *Z. marina* biomass [37], but the environmental microbiome in the seagrass meadow ecosystems will change with any type of interference (such as nutrient input) from kelp mariculture. Currently, studies are primarily focused on the mariculture economy or antibiotic residues in the environment [27, 29]. However, the influence of different mariculture on seagrass environmental microbiome remains unknown.

The seagrass-associated microbiomes in seawaters and sediments have been proven to be different [11, 38]. Our research interests in the microbiome of seawaters and rhizosphere sediments surrounding *Z. marina* were motivated by the effects of mariculture on seagrass and its microenvironment. Although mariculture affects the metabolism of the seagrass, little is known about the roles of microbial communities in this process. To fill this knowledge gap, we collected seawater and rhizosphere sediment samples from natural (N) sea, *Saccharina japonica* (SJ) culture, sea cucumber (SC) culture, and seagrass restoration (SR) zones, which were surrounding the *Z. marina*, and performed high-throughput sequencing of 16S and 18S rRNA and internal transcribed spacer (ITS) genes in addition to performing metagenomic sequencing. Our study had several aims: (1) investigate the differences in bacterial, eukaryotic, and fungal communities in the seawaters and rhizosphere sediments of the *Z. marina* in different mariculture zones; (2) analyze the metabolic pathways and functional genes using metagenomic sequencing; and (3) elucidate the potential influencing mechanisms of mariculture on the environmental microbiome surrounding *Z. marina*.

## Materials and methods

### Sampling and environmental parameters

Surrounding seawater and rhizosphere sediment samples of *Z. marina* were collected from natural and mariculture zones (N, SJ, SC and SR) in Yantai and Weihai of Shandong Province, China, in June 2021 (Fig. S1). The seawater samples from NS, SJ, SC and SR zones were named NSW, SJSW, SCSW and SRSW, respectively, and rhizosphere sediment samples of these four zones were named NRS, SJRS, SCRS and SRRS, respectively. At each sampling location, four replicate samples (~50 m apart each) were collected. One liter of seawater was filtered through 0.22-μm polycarbonate membranes (Millipore Corporation, Billerica, MA, USA) for the sequencing of bacterial, eukaryotic, and fungal communities. Ten liters of seawater was filtered through 0.22-μm polycarbonate membranes (Millipore Corporation, Billerica, MA, USA) for metagenomic sequencing. The *Z. marina* seagrass was shaken to remove the loose soil and scraped using a plastic spoon to collect the corresponding rhizosphere sediment samples. Membranes and rhizosphere sediment samples were frozen in liquid nitrogen immediately, and then stored at – 80 °C in the laboratory.

Nutrients in seawater samples, including nitrate (NO_3_^−^), nitrite (NO_2_^−^), ammonium (NH_4_^+^), phosphate (PO_4_^3−^), and dissolved silicon (SiO_3_^2−^), were measured using a nutrient auto-analyzer (AA3, Seal Analytical Ltd, UK) after filtering with 0.45-μm cellulose acetate membranes [39]. The mean (MD) and mid-value (D50) diameters of rhizosphere sediment were measured with a laser particle analyzer (Malvern Instruments, UK) after removing the large particles using a 2-mm sieve. The rhizosphere sediment samples were dried at 60 °C, sieved through 149-μm sieve and ground using a porcelain mortar for the detection of the following environmental parameters. For nutrients detection, 25 mL KCl (2 M) was added to 2.5 g rhizosphere sediment sample and shaken for 1 h. After filtering with 0.45-μm cellulose acetate membranes, NO_3_^−^, NO_2_^−^, and NH_4_^+^ concentrations were detected using a nutrient auto-analyzer (AA3, Seal Analytical Ltd, UK). For the measurement of sulfate (SO_4_^2−^), chloride (Cl^−^), and bromide (Br^−^), ultrapure water was added to the rhizosphere sediment at a ratio of 5:1 (w/w) and shaken for 1 h. The Cl^−^, Br^−^, and SO_4_^2−^ concentrations were detected using Ion Chromatograph (Dionex ICS-3000, USA) after filtered with 0.45-μm cellulose acetate membranes. For the detection of total carbon (TC), total organic carbon (TOC), and total organic nitrogen (TON), 3 mL hydrochloric acid (HCl, 1 M) was added to a brown bottle containing 2 g rhizosphere sediment, shaking for 1 h and discarding supernatant. Adding 1 M HCl into bottle until no bubbles generated, discarding the supernatant after shaking for 1 h. Three milliliters of ultrapure water was added into the brown bottle, mixed and dried in a 60 °C oven. The TC, TOC, and TON were detected using vario MICRO cube elemental analyzer (Elementar, Germany) [40].

### DNA extraction

Total DNA of seawater samples, including samples for 16S rRNA, 18S rRNA, ITS sequencing, and metagenomic sequencing, was extracted using E.Z.N.A.® Soil DNA Kit (Omega Bio-Tek, Georgia, USA) following the manufacturer’s protocol [41]. The DNA of rhizosphere sediment samples was extracted using the FastDNA^TM^ Spin Kit for Soil (MP Biomedicals, California, USA) [42]. All the DNA was dissolved in 30 μL DNase-free water to increase DNA concentration and sent to Majorbio Bio-Pharm Technology Co. Ltd. (Shanghai, China) for sequencing.

### Bacterial, eukaryotic, and fungal community structure analyses

The bacterial 16S rRNA gene was amplified using 338F and 806R primers (Table S1) [43]. The PCR reaction mixture (20 μl) contained 2× Pro Taq (10 μl), forward and reverse primers (4 μM), and template DNA (200 ng). PCR cycling conditions were as follows: 1 cycle of 3 min at 95 °C, 30 cycles of 30 s for denaturation at 95 °C, 30 s for annealing at 53 °C and 45 s for elongation at 72 °C, 1 cycle of 10 min at 72 °C. The amplified PCR products were purified using an AxyPrep DNA Gel Extraction Kit (Axygen Biosciences, CA, USA) and quantified using QuantiFluor™-ST (Promega, USA). Purified amplicons were paired-end sequenced (2 × 300 bp) on the Illumina MiSeq platform (Illumina, San Diego, USA) by Majorbio Bio-Pharm Technology Co. Ltd. (Shanghai, China). The eukaryotic 18S rRNA gene was amplified using TAReukFWD1F and TAReukREV3R primers (Table S1) [44], and the PCR condition was the same with 16S rRNA gene except for the 55 °C annealing temperature and 35 cycles. The fungal ITS gene was amplified using ITS1F and ITS2R primers (Table S1) [45], and the PCR conditions were the same with 18S rRNA gene amplification.

Raw data were filtered using QIIME2 (version 2022.2) [46] and then trimmed with FASTP to remove low-quality reads (< 100 bp) [47]. Paired-end sequences were joined with at least a 10-bp overlap and < 5% mistakes using FLASH [48]. Then, the amplicon sequence variants (ASVs) of 16S rRNA, 18S rRNA, and ITS were clustered using QIIME2 with 100% similarity. Barcodes, chimeras, primers, and low-quality reads were removed using the DADA2 plug-in within QIIME2 software [49]. The random rarefaction of each sample was performed to obtain an equal sequencing depth (i.e., to obtain the same minimum number of sample sequences). The taxonomic classification of the 16S rRNA gene sequence for all ASVs was analyzed against the Silva 138 16S rRNA database using a classify-sklearn classifier with a confidence threshold of 70%.

Alpha diversity indices, including Good’s coverage, Chao 1, and Shannon indices, were calculated using the MOTHUR to estimate the richness (Chao 1) and diversity (Shannon) of the bacterial, eukaryotic, and fungal communities [41]. The linear discriminant analysis effect size (LEfSe) was performed to assess the taxa enrichment in these four zones with linear discriminant analysis (LDA) values > 4, and taxa that were significantly higher in all four zones were set as the enriched taxa (biomarkers) [50]. The nonmetric multidimensional scaling (NMDS) analysis was used to evaluate ASVs differences. Canonical correspondence analysis (CCA) was used to analyze correlations between bacterial communities and environmental factors. Both the NMDS and CCA analyses were performed on R software (v3.3.1) using “vegan” package. The variation partition analysis (VPA) was performed on R software (v3.3.1) using “vegan” package to investigate the relative contribution of different environmental parameter groups to bacterial, eukaryotic, and fungal communities. The correlations between different environmental parameters as well as the bacterial, eukaryotic, and fungal taxa were calculated using Mantel test on R software (v3.3.1) using “vegan” and “ggcor” packages [51]. The co-occurrence network was analyzed on R software (v3.3.1) using “Hmisc” package and plotted using Gephi software (v0.9.2).

### Metagenomic sequencing and analysis

Metagenomic sequencing was performed on the Illumina HiSeq NovaSeq 6000 platform (2 × 150 bp paired-end). The raw reads were filtered using Fastp (v0.20.0) to remove low-quality reads (contain > 10% undefined bases, > 40% low-quality bases, or >15 bases adapter sequence). Clean data were obtained from each sample after filtration, ranging from 16.26 to 18.26 Gb. These high-quality reads were assembled using Megahit (v1.1.2), and contigs < 300 bp were removed [52]. The qualities of assemblies were assessed using Quast (v5.2.0) and were detailed in the Table S2. The genes were predicted using Prodigal (v2.6.3) and clustered to remove redundant sequences using CD-Hit at 90% identity and 90% coverage [53]. The reads per kilobase million (RPKM) was used to calculated the relative abundance of genes by SOAPaligner (v2.21) [54].

The initial paired-end reads were co-assembled using IDBA-UD [55]. Subsequently, these reads were mapped to the final assembled contigs with Burrows-Wheeler Alignment tool [56]. Contigs were grouped into metagenome assembled genomes (MAGs) based on empirical probabilistic distances of genome abundance and tetranucleotide frequency using program MetaBAT2 [57]. Completeness and contamination of MAGs were assessed using CheckM (v1.2.2) [58], and MAGs with a completeness above 80% and contamination lower than 10% were considered for further analysis. The assignment of MAGs taxonomy and the subsequently phylogenomic tree construction were conducted using GTDB-Tk tool (v2.3.2) based on GTDB (https://gtdb.ecogenomic.org) database [59]. Genome annotation of the MAGs was carried out using the RAST server (https://rast.nmpdr.org).

Representative sequences of non-redundant genes were annotated against the NR database (v20200604) in NCBI to obtained the taxonomy assignment using Diamond (v0.8.35) with an e-value cutoff of 10^−5^ (blastp) [60]. The Kyoto Encyclopedia of Genes and Genomes (KEGG) annotation was conducted against the KEGG database (version 94.2) using the Diamond software (v0.8.35) with an e-value cutoff of 10^−5^ [61]. ARGs annotation was conducted using Diamond (0.8.35) against the Comprehensive Antibiotic Resistance Database (CARD) with an e-value cutoff of 10^−5^ [62]. The relative contributions of each taxon to KEGG and CARD functions were calculated as described previously [63]. The LEfSe was performed to assess the enriched functions in these four zones with LDA values > 2. Functions that were significantly higher in all of these four zones were set as enriched functions (biomarkers).

### Statistical analyses

Unless otherwise stated, the difference in environmental parameters, the bacterial, eukaryotic, and fungal community (composition and α diversity), and the metagenomic analysis (KEGG and CARD analyses) between these four zones were tested using the Wilcoxon rank-sum test. The correlations between different environmental parameters and taxa of bacteria, eukaryota, and fungi were analyzed using the Spearman correlation test. All statistical analyses were performed using SPSS version 24.0 (SPSS Inc., Chicago, IL, USA). *P <* 0.05 and *P <* 0.01 were considered statistically significant.

## Results

### Environmental parameters

The environmental parameters of the seawater and rhizosphere sediment samples surrounding *Z. marina* are shown in Tables S3 and S4. Generally, the seawater samples of these four zones had a higher level of SiO_3_^2−^ and NH_4_^+^ concentrations (Table S3). The NSW samples presented the highest NH_4_^+^ (4.32 ± 0.46 μM) and lowest SiO_3_^2−^ (2.78 ± 0.16 μM) concentrations. The highest NO_2_^−^ and NO_3_^−^ concentrations were presented in the SJSW samples (0.46 ± 0.26 μM and 23.21 ± 22.00 μM), whereas the lowest levels of these two nutrients were detected in the SRSW samples (0.09 ± 0.02 μM and 0.12 ± 0.05 μM). In contrast, the highest PO_4_^3−^ concentration was detected in the SRSW samples (0.57 ± 0.20 μM), while the lowest concentration was present in the SJSW samples (0.26 ± 0.10 μM). The concentration of SiO_3_^2−^ was highest in the SJSW samples (16.91 ± 12.69 μM) but lowest in the NSW samples (2.78 ± 0.16 μM).

As for the rhizosphere sediments, the SJRS samples had relatively higher levels of NH_4_^+^ (1.19 ± 0.24 μmol/kg), NO_2_^−^ (5.51 ± 4.59 μmol/kg), NO_3_^−^ (22.70 ± 12.20 μmol/kg), Cl^−^ (222.96 ± 96.120 mmol/kg), Br^−^ (0.27 ± 0.15 mmol/kg), and SO_4_^2−^ (9.11 ± 4.49 mmol/kg) concentrations in addition to particle diameters (D50 and MD, 170.49 ± 18.96 μm and 184.57 ± 15.88 μm, respectively); however, the highest concentrations of TON (0.30% ± 0.05%), TC (3.04% ± 0.47%), and TOC (2.66% ± 0.14%) were present in SCRS samples (Table S4). The lowest concentrations of those environmental parameters were present in the NRS (NO_3_^−^, TC, and TOC) and SRRS (NH_4_^+^, Cl^−^, Br^−^, SO_4_^2−^, and MD) samples.

### Bacterial, eukaryotic, and fungal community compositions

The sequencing of 16S and 18S rRNA and ITS genes was performed on seawater and rhizosphere sediment samples from four diverse zones surrounding *Z. marina*. For the 16S rRNA sequencing, we obtained 966,249 sequences after “denoising” the system using DADA2, ranging from 19,831 to 46,237. The average length of these samples was 417 bp. A total of 32,182 ASVs were assigned after random rarefaction. Good’s coverage values of all samples ranged from 98.39 to 100.00%, indicating a good representativeness of the bacterial communities in these samples (Table S5). Generally, a higher ASV numbers were found in the rhizosphere sediment samples versus the seawater samples (*P* = 0.021 for all the four zones), indicating a higher bacterial community richness in the rhizosphere sediments. The alpha indices also showed that the richness (Chao 1 index) and diversity (Shannon index) of the bacterial communities in the rhizosphere sediment of these four zones were higher than in seawater samples (*P* = 0.030 for all the four zones, Fig. S2A and D). Besides, the richness (Chao 1 index) and diversity (Shannon index) of the bacterial communities in the SJSW samples were higher than in the NSW and SRSW samples (*P* = 0.030 for both, Fig. S2A and D).

For the 18S rRNA sequencing, we obtained 1,971,641 sequences after applying a “denoising” protocol using DADA2 that ranges from 32,051 to 84,069. The average length of these samples was 378 bp. After random rarefaction, a total of 19,123 ASVs were assigned. The Good’s coverage values of all samples ranged from 99.50 to 100.00%, indicating a good representativeness of the eukaryotic communities (Table S5). The ASV numbers in the SRRS samples were higher than in the SRSW samples (*P* = 0.021), but no significant differences in the ASV numbers between the seawater and rhizosphere sediment samples in the NS, SJ, and SC zones (*P* = 0.248, 0.149 and 0.773, respectively) were detected. The alpha indices also indicated that only the eukaryotic community in the SRRS samples had a higher richness (Chao 1) and diversity (Shannon) than in the SRSW samples (Fig. S2B and E). Furthermore, the eukaryotic community of SRSW samples had the minimum richness and diversity among the seawater samples of all these four zones (Fig. S2B and E), but no significant differences were found between the rhizosphere sediment samples (*P* > 0.05).

For ITS gene sequencing, we obtained 1,531,903 sequences after applying a “denoise” protocol using DADA2, ranging from 26,120 to 71,292. The average length of these samples was 242 bp. After random rarefaction, a total of 11,642 ASVs were assigned. Good’s coverage values of all samples ranged from 99.86 to 100.00%, indicating good representativeness of the fungal communities (Table S5). The ASV numbers in NRS and SRRS samples were higher than those in corresponding seawater samples (*P* = 0.021 for both), but no such difference was found in the SJ and SC zones (*P* = 0.248 and 0.083, respectively). The richness (Chao 1) of the fungal community was also higher in the NRS and SRRS samples than in seawater samples, but not in the SJ and SC zones (Fig. S2C). As for the diversity (Shannon), only the SRRS samples were lower than the SJRS and SCRS samples (*P* = 0.030 for both).

The metagenomic results showed that bacteria were the predominant taxa both in the seawater and rhizosphere sediment samples (94.76% ± 2.78% and 97.02% ± 1.46%), followed by the eukaryota (2.59% ± 2.43% and 1.16% ± 1.12%), archaea (1.12% ± 0.50% and 1.55% ± 0.40%), and viruses (1.69% ± 0.93% and 0.19% ± 0.12%) (Fig. S3). The proportion of fungi was relatively lower both in seawater (0.10% ± 0.15%) and rhizosphere sediment samples (0.03% ± 0.02%). Eukaryota was highest in the SRSW samples (5.72% ± 3.04%) but lowest in the SRRS samples (0.46% ± 0.24%). Viruses were relatively higher in seawaters (1.53% ± 0.84%) especially in the SJSW samples (2.52% ± 0.20%). On the contrary, archaea were more abundant in rhizosphere sediment samples (1.51% ± 0.40%) than in seawater samples (1.06% ± 0.50%).

**Fig. 1:**
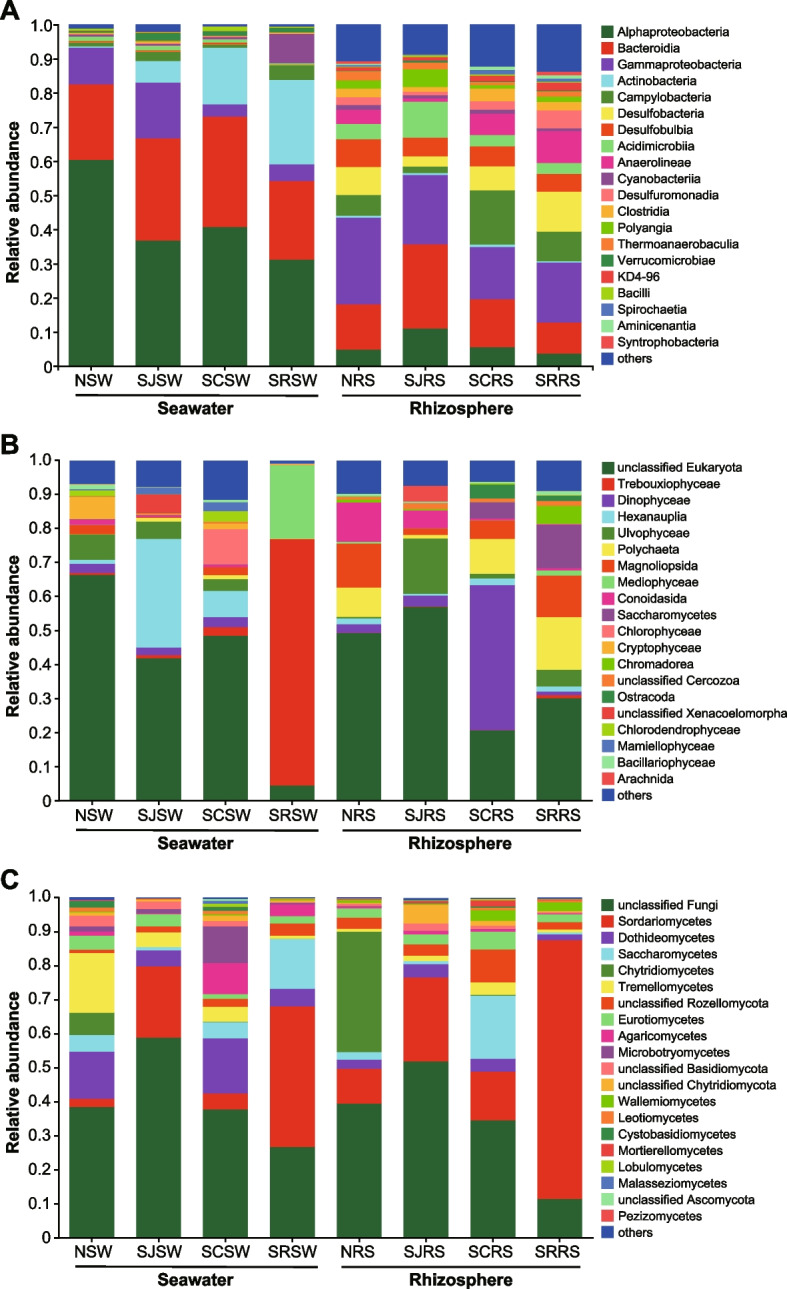
The bacterial (**A**), eukaryotic (**B**), and fungal (**C**) community composition in seawater and rhizosphere sediment samples from the N, SJ, SC, and SR zones at class level. The top 20 classes are shown

**Fig. 2:**
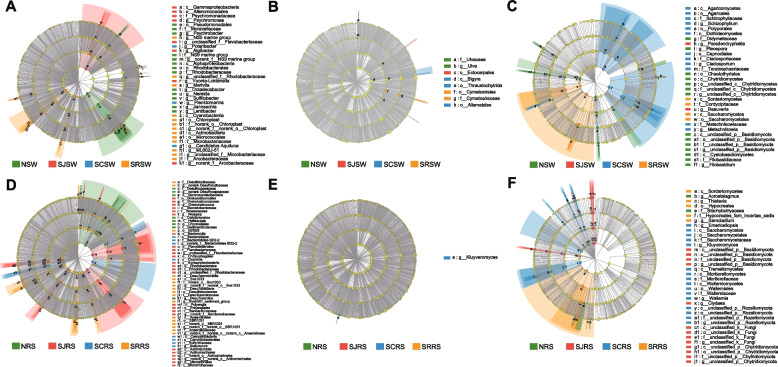
The LEfSe analysis for the bacterial, eukaryotic, and fungal communities in the seawater (**A**, **B**, and **C**) and rhizosphere sediment (**D**, **E**, and **F**) samples from the N, SJ, SC, and SR zones. Taxa that significantly higher in all of these four zones are set as the enriched taxa, and the taxa with LDA > 4 are shown

Obvious differences in the bacterial community between the seawater and rhizosphere sediment samples at class level in these four zones were found (Fig. 1A). In the seawater samples, *Alphaproteobacteria* (42.30% ± 12.56%) was the predominant group followed by *Bacteroidia* (26.87% ± 7.55%), *Actinobacteria* (11.97% ± 10.46%), and *Gammaproteobacteria* (8.86% ± 6.08%). However, *Gammaproteobacteria* (19.65% ± 4.19%) was the predominant bacteria in these rhizosphere sediment samples, followed by *Bacteroidia* (15.26% ± 6.40%), *Campylobacteria* (namely *Epsilonproteobacteria*, 8.10% ± 6.00%), *Desulfobacteria *(7.49% ± 3.97%), and *Alphaproteobacteria* (6.31% ± 3.35%). The results of the ELfSe analysis showed that *Alphaproteobacteria* (60.38% ± 3.35%) was the bacteria that enriched in the NSW samples when compared with the seawater samples from the other three zones, and the *Sulfitobacter* (23.02% ± 0.87%) may be the main contributor to the enrichment (Fig. 2A and S4A). *Gammaproteobacteria* (16.27% ± 6.20%) were significantly enriched in the SJSW samples, and *Psychromonas* (2.98% ± 2.18%) was the enriched genus of this class. *Cyanobacteria* (8.65% ± 3.17%) and *Actinobacteria* (24.66% ± 2.63%) were mainly enriched in the SRSW samples, and unclassified Chloroplast (6.94% ± 2.52%) and ML602J-51 (13.23% ± 2.38%) were the main contributors to these two classes. For the rhizosphere sediment samples, *Gammaproteobacteria* (25.34% ± 2.19%) were enriched in the NRS samples, and families of *Desulfocapsaceae* (6.67% ± 0.76%), *Halieaceae* (4.15% ± 0.55%), and *Sedimenticolaceae* (2.39% ± 0.40%) were the main contributors (Fig. 2D). However, only the norank *Desulfocapsaceae* (4.95% ± 0.61%) were found enriched at the genus level (Fig. 2D and S4D). *Bacteroidia* (24.73% ± 3.00%), *Alphaproteobacteria* (10.99% ± 1.31%), *Polyangia* (5.09% ± 0.99%), and *Acidimicrobiia* (10.50% ± 1.75%) were all enriched in the SJRS samples, and unclassified *Flavobacteriaceae* (4.90% ± 1.00%), unclassified *Rhodobacteraceae* (3.48% ± 1.39%), norank *Sandaracinaceae* (4.75% ± 0.38%), and norank *Actinomarinales* (4.64% ± 0.61%) were the enriched genera for the corresponding classes described above. *Clostridia* (3.71% ± 0.81%) and *Campylobacteria* (15.91% ± 5.08%) were the two enriched classes in the SCRS samples, and *Sulfurovum* (15.12% ± 5.40%) was the enriched genus of the *Campylobacteria* class. For the SRRS samples, *Desulfuromonadia* (5.29% ± 1.11%), *Desulfobacteria* (11.71% ± 4.67%), and *Anaerolineae* (9.36% ± 2.52%) were significantly enriched, and the norank Sva1033 (5.08% ± 1.09%), Sva0081 sediment group (5.96% ± 2.80%), and norank SBR1031 (2.97% ± 1.67%) were the corresponding contributing genera.

No consistent trend in the eukaryotic community was found, and the predominant classes and genera were different between these four zones in addition to between the seawater and rhizosphere sediment samples (Fig. 1B, S4B and E). *Ulvophyceae* (7.33% ± 3.58%), *Hexanauplia* (31.91% ± 26.34%), *Chlorophyceae* (10.26% ± 8.43%), and *Trebouxiophyceae* (72.57% ± 12.78%) were the predominant eukaryota at the class level in the NSW, SJSW, SCSW, and SRSW samples, while *Magnoliopsida* (12.97% ± 12.63%), *Ulvophyceae* (16.23% ± 24.89%), *Dinophyceae* (42.59% ± 38.90%), and *Polychaeta* (15.40% ± 25.51%) were the predominate classes in the NRS, SJRS, SCRS, and SRRS samples (Fig. 1B). For the enrichment analysis (using LEfSe), *Bigyra* was found to be enriched in the SCSW samples (1.62% ± 0.64%), but no significantly enriched genus was observed (Fig. 2B). Only the *Ulva* genus was found to be enriched in the NSW samples (6.39% ± 3.43%). For the rhizosphere sediment samples, only the genus *Kluyveromyces* (0.43% ± 0.22%) was found to be enriched in the SCRS samples (Fig. 2E).

In addition to the significant difference between the seawater and rhizosphere sediment samples, the composition of the fungal community in these four zones also has region-specific characteristics at the class level (Fig. 1C). Generally, *Sordariomycetes* was the predominate fungi in both seawater and rhizosphere sediment samples in the SJ (20.95% ± 31.99% and 24.62% ± 8.75%, respectively) and SR (41.31% ± 36.24% and 76.10% ± 10.94%, respectively) zones. Besides, *Saccharomycetes* also has a high proportion in the SRSW samples (14.61% ± 9.97%). For the N zone, *Dothideomycetes* (13.83% ± 2.96%) and *Tremellomycetes* (17.53% ± 19.08%) in the NSW samples and *Chytridiomycetes* (35.34% ± 17.11%) and *Sordariomycetesin* (10.32% ± 4.25%) in the NRS samples were predominant. *Dothideomycetes* (16.10% ± 6.48%), *Microbotryomycetes* (10.72% ± 14.61%), and *Agaricomycetes* (9.17% ± 4.55%) were predominant in the SCSW samples while *Saccharomycetes* (18.54% ± 6.08%), *Sordariomycetes* (14.30% ± 5.59%), and unclassified *Rozellomycota* (9.69% ± 4.07%) were predominant in the SCRS samples. For the enrichment analysis (using LEfSe) of the seawater samples, *Chytridiomycetes* (6.55% ± 3.10%), unclassified *Basidiomycota* (3.13% ± 2.69%), and *Cystobasidiomycetes* (1.82% ± 1.30%) were found to have been enriched in the NSW samples, and two unclassified genera in *Basidiomycota* (3.13% ± 2.69%) and *Chytridiomycetes* (6.55% ± 3.10%) were the main contributors of the corresponding two classes (Fig. 2C). Only the genus *Paradendryphiella* (1.18% ± 0.51%) was found to have been enriched in the SJSW samples. *Agaricomycetes* and *Dothideomycetes* were found to have been enriched in the SCSW samples, and *Schizophyllum* (5.59% ± 3.56%) and *Cladosporium* (7.63% ± 4.14%) were the main contributors to the corresponding two classes described above. *Sordariomycetes* and *Saccharomycetes* were enriched in the SRSW samples, and no enriched genus was found in these two classes. As for the rhizosphere sediment samples, the genus *Acrostalagmus* (0.12% ± 0.11%) was found to have been enriched in the NRS samples, but no enrichment result was found at the class level (Fig. 2F). Two unclassified classes of *Basidiomycota* (2.23% ± 0.79%) and *Chytridiomycota* (5.43% ± 5.15%) were enriched in the SJRS samples, and two unclassified genera of these two classes were the main contributors to the enrichment. In the SCRS samples, *Saccharomycetes*, *Wallemiomycetes* (3.12% ± 1.46%), unclassified *Rozellomycota*, *Tremellomycetes* (3.65% ± 3.52%), and *Mortierellomycetes* (1.73% ± 2.96%) were found to be enriched, and *Kluyveromyces* (15.44% ± 4.60%), *Wallemia* (3.12% ± 1.46%), and unclassified *Rozellomycota* (9.69% ± 4.07%) were the main contributors to the enrichment for the former three classes. *Sordariomycetes* were enriched in the SRRS samples, and *Thielavia* (0.10% ± 0.07%) and *Sarocladium* (73.44% ± 11.11%) were the two enriched genera of *Sordariomycetes*.

To seek the diversity and potential functions of uncultured novel bacteria, we constructed 109 high-quality MAGs with a contaminant threshold of 10% and a completeness threshold of 80% (Table S6). These MAGs included 24 Alphaproteobacteria (mainly the Rhodobacteraceae), 12 Gammaproteobacteria, 36 Bacteroidia (all the Flavobacteriaceae), 21 Verrucomicrobiae (potential probiotics), and some other bacteria such as Desulfobacteria. Interestingly, ~87% MAGs were obtained from the seawater samples, which was ~6.7-fold higher than the rhizosphere sediment samples (Fig. S5), although these samples had similar clean data (16.26 to 18.26 Gb). The results of the phylogenomic tree analysis suggested that the MAGs from NSW samples were mostly Alphaproteobacteria and Bacteroidia, while MAGs from SRSW were mostly Alphaproteobacteria. MAGs in the SJSW and SCSW had higher diversity than the former two sample groups, and most of them belonged to Verrucomicrobiae and Bacteroidia (Fig. S5).

### Interactions and diversity of bacterial, eukaryotic, and fungal communities

**Fig. 3:**
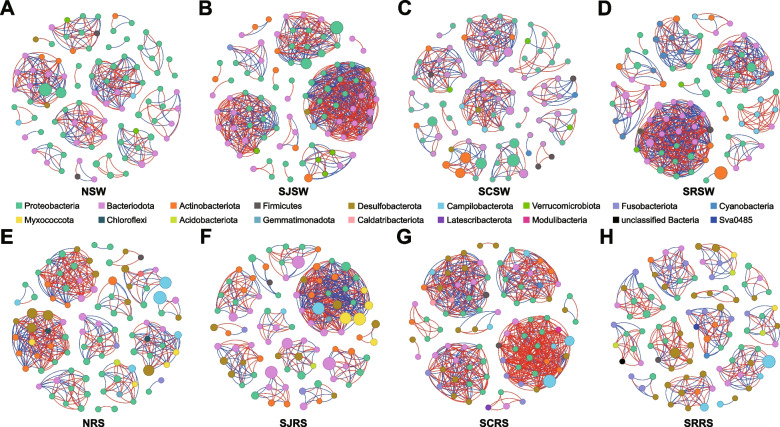
Co-occurrence network diagram of bacterial communities at ASV level in seawater (**A**, **B**, **C**, and **D**) and rhizosphere sediment (**E**, **F**, **G**, and **H**) samples from the N, SJ, SC, and SR zones. ASVs of the top 100 abundance in each sample are selected. The colors of spots indicate different bacterial phyla, and lines between spots indicate correlations between ASVs. Red lines, positive correlations. Blue lines, negative correlations

The co-occurrence network analysis was performed using the top 100 ASVs to study the interactions of bacterial, eukaryotic, and fungal communities in these four zones (Fig. 3, S6, and S7). The nodes in the co-occurrence network analysis were similar and ranged from 92 to 97, 90 to 93, and 94 to 97 in bacterial, eukaryotic, and fungal communities, respectively (Table 1). The edges in the bacterial community in the SJSW and SRSW samples were higher when compared with the NSW samples (1.9- and 1.7-fold, respectively), and the proportions of positive and negative correlations were equal (Fig. 3A, B, D). The SCRS samples also had a higher edge number than the NRS samples (1.5-fold), which was mainly due to positive correlations (Fig. 3G). As for the eukaryotic community, no significant difference in the number of edges between these four zones was found, but the proportions of positive correlations in SJSW (89.69%) and SRSW (72.46%) samples were higher than in NSW (52.51%) samples (Fig. S6B and D). When compared with the eukaryotic community in the NRS samples, only the SCRS samples had a higher edge number, and the proportions of positive correlations in the SJRS, SCRS, and SRRS samples (65.25%, 69.13%, and 75.72%, respectively) were lower than in the NRS samples (88.15%, Fig. S6E-H). The edge numbers in the fungal communities of the SJSW and SRSW samples were higher than in the NSW samples, and the SJSW, SCSW, and SRSW samples showed a higher proportion of positive correlations (88.86%, 83.24%, and 97.35%, respectively) than the NSW samples (67.83%, Fig. S7A-D). The edge numbers of fungal communities in the rhizosphere sediment were similar except for the SCRS samples (60.00%), which was lower than the other three zones (Fig. S7E-H).

**Table 1:** The properties of the co-occurrence network for bacterial, eukaryotic, and fungal communities in seawater and rhizosphere sediment samples of natural and mariculture zones

Communities	Properties	NSW	SJSW	SCSW	SRSW	NRS	SJRS	SCRS	SRRS
Bacterial community	Nodes	96	95	96	92	97	92	92	94
	Edges	331	633	333	558	431	463	658	338
	Positive correlations	179	333	176	287	238	229	414	188
	Negative correlations	152	300	157	271	193	234	244	150
Eukaryotic community	Nodes	95	94	91	95	93	90	95	93
	Edges	354	359	315	276	329	305	460	243
	Positive correlations	186	322	182	200	290	199	318	184
	Negative correlations	168	37	133	76	39	106	142	59
Fungal community	Nodes	95	95	97	94	97	96	94	94
	Edges	230	368	179	415	288	298	260	218
	Positive correlations	156	327	149	404	211	253	156	171
	Negative correlations	74	41	30	11	77	45	104	47

The NMDS and CCA analyses were performed to investigate the dissimilarity of bacterial, eukaryotic, and fungal communities (Figs. S8 and S9). The results showed significant differences in the bacterial, eukaryotic, and fungal communities between seawater and rhizosphere sediment samples, particularly in the bacterial community (red and blue spots, Fig. S8). The Anosim analysis also confirmed significant differences in these three communities between the seawater and rhizosphere sediment samples (*R* = 0.999, 0.962 and 0.925 for bacterial, eukaryotic, and fungal communities, respectively, *P* = 0.001 for all of them). The bacterial, eukaryotic, and fungal communities in the NSW, SJSW, and SCSW were similar, but those in the SRSW samples differed from the other three zones. However, no such significant trend was observed in the rhizosphere sediment samples for the bacterial, eukaryotic, and fungal communities.

### Correlations between environmental parameters and communities of bacteria, eukaryotes, and fungi

When combined with the environmental parameters, the CCA revealed that the correlations between environmental factors and communities of bacteria, eukaryotes, and fungi were similar both in seawater and rhizosphere sediment samples (Fig. S9). The NO_3_^−^, NO_2_^−^, and SiO_3_^2−^ concentrations were the main environmental factors that affected the bacterial, eukaryotic, and fungal communities in the SJSW samples, and NH_4_^+^ and PO_4_^3−^ concentrations affected the bacterial, eukaryotic, and fungal communities in the NSW and SRSW samples, respectively (Fig. S9A-C). As for the rhizosphere sediment samples, NO_3_^−^, NO_2_^−^, NH_4_^+^, Br^−^, and MD were the important factors for the SJRS samples, and TOC concentration may have affected the bacterial, eukaryotic, and fungal communities in the SCRS samples (Fig. S9D-F).

To evaluate the influence of different environmental parameter groups on those communities, N (including NO_3_^−^, NO_2_^−^, and NH_4_^+^), P (PO_4_^3−^) and Si (SiO_3_^2−^) groups for seawater samples and C (including TC and TOC), N (including NO_3_^−^, NO_2_^−^, NH_4_^+^, and TON), and D (including MD and D50) groups for rhizosphere sediment samples were chosen to conduct the VPA analysis as shown in Fig. S10. The N group contributed the highest variation (43.67%) for the total bacterial community in the seawater samples, which was the same for the eukaryotic community (32.62%). However, the contribution from the N group to the fungal community in seawater samples was lower at 8.20% in addition to the contributions from PO_4_^3−^ and SiO_3_^2−^. PO_4_^3−^ contributed more to the eukaryotic community of seawater samples than to the bacterial and fungal communities (Fig. S10A-C). As for the rhizosphere sediment samples, the C group was the main contributor (29.25%) for the bacterial community, followed by the D group a with 16.73% contribution (Fig. S10D). All of the C, N, and D groups contributed less to the eukaryotic communities in the rhizosphere sediment samples (8.81%, 2.55%, and 3.08%, respectively, Fig. S10E). The N group was the main contributor to fungi and accounted for 14.48% of the total community (Fig. S10F).

**Fig. 4:**
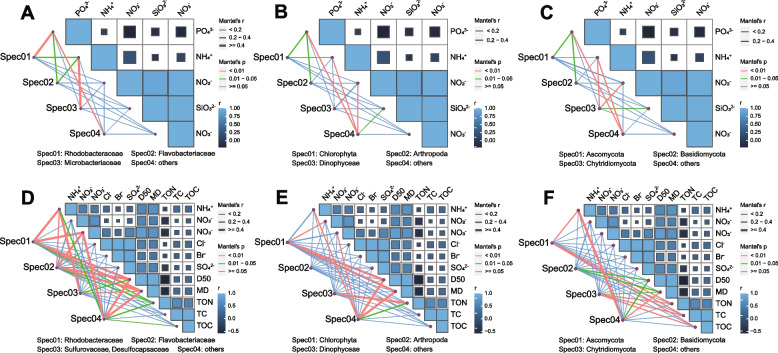
The Mantel test of bacterial, eukaryotic, and fungal communities in seawater (**A**, **B**, and **C**) and rhizosphere sediment (**D**, **E**, and **F**) samples from the N, SJ, SC, and SR zones. ASVs of the top 50 abundance in each sample are selected. Families (bacteria) or phyla (eukaryota and fungi) of top three abundance are merged respectively as Spec01, Spec02, and Spec03. Line colors and thickness represent the *P* and R values of the Mantel test

The Mantel test was performed to analyze the correlations between different environmental factors and elucidate their interactions with distinct microorganism groups of the bacterial, eukaryotic, and fungal communities in the seawater and rhizosphere sediment samples (Fig. 4). Significantly positive correlations were observed in the seawater samples between NO_3_^−^, NO_2_^−^, and SiO_3_^2−^ (Fig. 4A–C, *P* = 0.001 for all, Pearson’s correlation test). For the rhizosphere sediment samples, significantly positive correlations were observed between particle diameter (D50 and MD) and some other environmental parameters, including NH_4_^+^ (*P* = 0.003 and 0.014), NO_2_^−^ (*P* = 0.047 and 0.025), NO_3_^−^ (*P* = 0.002 and 0.001), Cl^−^ (*P* = 0.055 and 0.044), and SO_4_^2−^ (*P* = 0.032 and 0.015) (Fig. 4D–F, Pearson’s correlation test). Besides, significantly positive correlations were also observed between NO_2_^−^ and NO_3_^−^ (*P* = 0.003), Cl^−^ and Br^−^ (*P* = 0.001), SO_4_^2−^ and NO_3_^−^ (*P* = 0.001), SO_4_^2−^ and Cl^−^ (*P* = 0.001), SO_4_^2−^ and Br^−^ (*P* = 0.001), D50 and MD (*P* = 0.001), TON and TC (*P* = 0.009), TON and TOC (*P* = 0.027), and TC and TOC (*P* = 0.001) in the rhizosphere sediment samples (Fig. 4D–F, Pearson’s correlation test). A significantly negative correlation was only found in the rhizosphere sediment samples between D50 and TC (*P* = 0.026, Pearson correlation test).

The top 50 ASVs of the bacterial community in seawater samples were grouped into *Rhodobacteraceae* (18 ASVs), *Flavobacteriaceae* (13 ASVs), *Microbacteriaceae* (6 ASVs), and others (13 ASVs) clusters at the family level. All three bacterial clades (except for the “others” clade) exhibited significant correlations with the NH_4_^+^ concentration (*P* = 0.003, 0.021, and 0.001), particularly for the *Microbacteriaceae* family (Fig. 4A). Besides, both *Rhodobacteraceae* and *Flavobacteriaceae* were found to be significantly correlated with the PO_4_^3−^ concentration (*P* = 0.001 and 0.023). No significant correlation was observed between those four clusters and NO_2_^−^, NO_3_^−^, and SiO_3_^2−^concentrations (Fig. 4A). Among the rhizosphere sediment samples, the top 50 ASVs of bacterial community were grouped into the *Rhodobacteraceae* (14 ASVs), *Flavobacteriaceae* (13 ASVs), *Sulfurovaceae, Desulfocapsaceae* (three ASVs, related to sulfur metabolism), and others (20 ASVs) clusters at the family level. Significant correlations between these four bacterial clusters and environmental parameters including NH_4_^+^ (*P* = 0.001, 0.003, 0.001, and 0.012), D50 (*P* = 0.001, 0.001, 0.044, and 0.001), and MD (*P* = 0.001, 0.001, 0.006 and 0.001) were found. The NO_3_^−^ (*P* = 0.010, 0.002, and 0.007) and TON (*P* = 0.020, 0.021, and 0.017) concentrations also significantly correlated with bacterial clusters, except for the *Sulfurovaceae* and *Desulfocapsaceae* clade.

In seawater and rhizosphere sediment samples, the top 50 ASVs of the eukaryotic community were grouped into *Chlorophyta* (10 ASVs), *Arthropoda* (7 ASVs), *Dinophyceae* (*Alveolata* clade, 5 ASVs), and others (28 ASVs) clusters at the phylum level. The number of significant correlations between environmental parameters and eukaryotic clusters was less than those in the bacterial community both in the seawater and rhizosphere sediment samples (Fig. 4B, E). Significant correlations were observed between *Chlorophyta* and the concentrations of PO_4_^3−^ (*P* = 0.027) and NH_4_^+^ (*P* = 0.035) in addition to between *Arthropoda* and PO_4_^3−^ (*P* = 0.011) concentrations in seawater samples (Fig. 4B). The *Chlorophyta* in the rhizosphere sediment samples significantly correlated with NO_2_^−^ (*P* = 0.007), D50 (*P* = 0.002), and MD (*P* = 0.001), while *Dinophyceae* only significantly correlated with TON (*P* = 0.010, Fig. 4E). No significant correlations were found between *Dinophyceae* and environmental factors in seawater samples. In addition, no significant correlations were found between *Arthropoda* and environmental factors in the rhizosphere sediment samples.

In seawater and rhizosphere sediment samples, the top 50 ASVs of the fungal community were grouped into *Ascomycota* (13 ASVs), *Basidiomycota* (8 ASVs), *Chytridiomycota* (three ASVs), and others (26 ASVs) clusters at the phylum level. The number of significant correlations between environmental parameters and fungal clusters was less than in the bacterial community both in the seawater and rhizosphere sediment samples, which was similar to the eukaryotic community (Fig. 4C, F). *Ascomycota* and *Basidiomycota* in seawater samples only correlated significantly with PO_4_^3−^ concentration (*P* = 0.026 and 0.049), and *Chytridiomycota* in seawater samples correlated with NH_4_^+^ (*P* = 0.001), NO_2_^−^ (*P* = 0.010), and SiO_3_^2−^ (*P* = 0.046) concentrations (Fig. 4C). As for the rhizosphere sediment samples, both *Ascomycota* and *Chytridiomycota* were significantly correlated with TON (*P* = 0.001 and 0.001), TC (*P* = 0.004 and 0.001), and TOC (*P* = 0.010 and 0.002) concentrations. Besides, *Ascomycota* also correlated significantly with NH_4_^+^ (*P* = 0.010), and *Chytridiomycota* also significantly correlated with MD (*P* = 0.049). *Basidiomycota* in rhizosphere sediment samples correlated significantly with particle diameters (D50 and MD, *P* = 0.040 and 0.013) as shown in Fig. 4F.

### Metagenome and metabolism analysis

The corresponding metagenomic sequencing (32 samples) was performed on seawater and rhizosphere sediment samples. The results of KEGG functions showed that the global and overview maps of metabolism (such as metabolic pathways, biosynthesis of secondary metabolites, biosynthesis of amino acids), nucleotide metabolism (both purine and pyrimidine metabolism), and genetic information processing (such as aminoacyl-tRNA biosynthesis, ribosome) were higher in seawater while the global and overview maps of metabolism (such as microbial metabolism in diverse environments, carbon metabolism), signal transduction (two-component system), cellular community of cellular processes (quorum sensing), carbohydrate metabolism (such as glycolysis/gluconeogenesis, citrate cycle, pyruvate metabolism), and energy metabolism (such as oxidative phosphorylation, methane metabolism) were higher in rhizosphere sediments (Fig. S11C, *P* = 0.001).

**Fig. 5:**
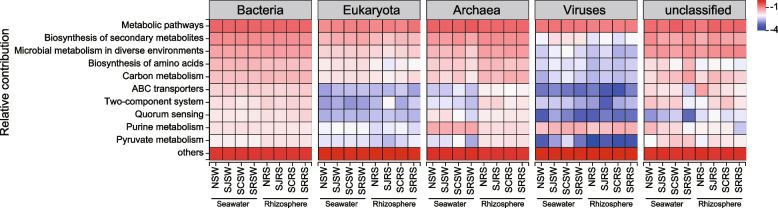
The KEGG functions in bacterial, eukaryotic, archaeal, and viral communities in seawater and rhizosphere sediment samples from the N, SJ, SC, and SR zones. The top 10 KEGG functions are shown

The contribution of various species to the KEGG functions in seawater and rhizosphere sediment samples was evaluated to investigate the different roles of bacteria, eukaryota, archaea, and viruses in terms of the KEGG functions in seawater and rhizosphere sediment samples (Fig. 5). The main contributor to biosynthesis of secondary metabolites, microbial metabolism in diverse environments, biosynthesis of amino acids, and carbon metabolism was bacteria, followed by archaea and eukaryota. However, the contribution of viruses to those functions was lower especially in the rhizosphere sediment samples (Fig. 5). As for the functions of ABC transporters, two-component system, quorum sensing, and pyruvate metabolism, bacteria were still the main contributors. Interestingly, viruses contribute more to purine and pyrimidine metabolism, homologous recombination, mismatch repair, DNA replication, RNA degradation, and one carbon pool by folate than the bacterial, eukaryotic, and fungal communities (Fig. S12). Archaea were found to play essential roles in the rhizosphere sediment samples with higher contributions than in seawater samples for most of these KEGG functions (Fig. S12). However, the contributions of archaea to purine and pyrimidine metabolism, mismatch repair, DNA replication, one carbon pool by folate, and nucleotide excision repair were opposite and were higher in the seawater samples than in rhizosphere sediment samples (Fig. 5 and S12). The contribution of archaea to carbohydrate metabolism in the SRSW samples was lower than those in the other seawater samples and rhizosphere sediment samples, such as pyruvate, butanoate, propanoate, citrate cycle, pentose phosphate pathway, glyoxylate, and dicarboxylate metabolism.

**Fig. 6:**
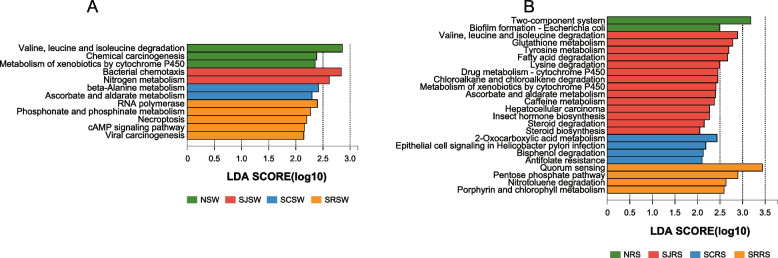
The LDA analysis of the KEGG functions in seawater (**A**) and rhizosphere sediment (**B**) samples from the N, SJ, SC, and SR zones. Functions that significantly higher in all of these four zones are set as enriched functions, and KEGG functions with LDA > 2 are shown

The LEfSe was performed to analyze the significantly enriched KEGG pathways (level 3) in the seawater and rhizosphere sediment samples from the N, SJ, SC, and SR zones (Fig. 6). For the seawater samples, valine, leucine, and isoleucine degradation (0.81% ± 0.01%) and chemical carcinogenesis (0.12% ± 0.01%) were enriched in the NSW samples, while bacterial chemotaxis (0.25% ± 0.04%) and nitrogen metabolism (0.36% ± 0.02%) were enriched in the SJSW samples. The β-alanine (0.29% ± 0.01%) and ascorbate and aldarate metabolic pathways (0.20% ± 0.01%) in the SCSW were enriched, whereas some pathways involved in cellular processes were enriched in the SRSW samples (RNA polymerase, phosphonate and phosphinate metabolism, necroptosis, cAMP signaling pathway, and viral carcinogenesis with 0.25% ± 0.01%, 0.10% ± 0.01%, 0.14% ± 0.01%, 0.03% ± 0.01%, and 0.07% ± 0.01%, respectively, Fig. 6A). As for the rhizosphere sediment samples, two-component system (2.01% ± 0.04%) and biofilm formation *Escherichia coli* (0.35% ± 0.01%) were enriched in the NRS samples, which were involved in signal transduction and cellular processes (Fig. 6B). Amino acid metabolism (glutathione, tyrosine, lysine, valine, leucine, and isoleucine with 0.48% ± 0.01%, 0.28% ± 0.01%, 0.44% ± 0.01%, and 0.77% ± 0.02%, respectively), lipid metabolism (fatty acid degradation and steroid biosynthesis with 0.58% ± 0.02% and 0.03% ± 0.01%, respectively), xenobiotics metabolism (steroid degradation, metabolism of xenobiotics by cytochrome P450, drug metabolism–cytochrome P450, and chloroalkane and chloroalkene degradation with 0.05% ± 0.01%, 0.10% ± 0.01%, 0.11% ± 0.01%, and 0.16% ± 0.01%, respectively), and some other metabolic pathways (ascorbate and aldarate metabolism, caffeine metabolism, and insect hormone biosynthesis with 0.15% ± 0.01%, 0.01% ± 0.01%, and 0.06% ± 0.01%, respectively) were enriched in the SJRS samples. Pathways related to metabolism (2-oxocarboxylic acid metabolism and bisphenol degradation with 0.79% ± 0.01% and 0.01% ± 0.01%, respectively) and human diseases (epithelial cell signaling in *Helicobacter pylori* infection and antifolate resistance with 0.05% ± 0.01% and 0.15% ± 0.01%, respectively) were enriched in the SCRS samples. The quorum sensing pathway (1.92% ± 0.09%) in addition to several metabolic pathways, including the pentose phosphate pathway (0.75% ± 0.03%), nitrotoluene degradation (0.17% ± 0.01%), and porphyrin and chlorophyll metabolism (0.58% ± 0.01%), was significantly enriched in the SRRS samples (Fig. 6B).

### Nitrogen and sulfur metabolism

**Fig. 7:**
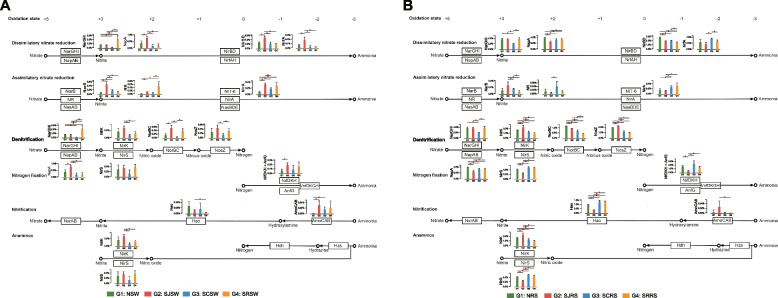
The nitrogen metabolism in seawater (**A**) and rhizosphere sediment (**B**) samples from the N, SJ, SC, and SR zones. Wilcoxon rank-sum test is used to analyze the difference in nitrogen metabolic genes in the seawater and rhizosphere sediment samples between the N, SJ, SC, and SR zones. *P* < 0.05 is marked with “*”

Considering that the nitrogen metabolism pathway was significantly enriched in the SJRS samples, we analyzed the dissimilatory reduction, assimilatory nitrate reduction, denitrification, nitrogen fixation, nitrification, and anammox pathways in the seawater and rhizosphere sediment samples of those four zones (Fig. 7). Generally, the levels of most nitrogen metabolic proteins, especially those related to the dissimilatory nitrate reduction, denitrification, and nitrogen fixation pathways, were higher in the rhizosphere sediment samples than in seawater samples (Fig. S13). When compared with the NSW, SCSW, and SRSW samples, the SJSW samples had a higher proportion of dissimilatory nitrate reduction and assimilatory nitrate reduction metabolism. The abundances of the peptidoglycan-associated protein NapA (0.87% ± 0.15%), nitrate reductase NirBD (5.35% ± 0.98%), and the cytochrome c nitrite reductase NrfA (0.23% ± 0.07%) proteins, which function in the dissimilatory reduction of nitrate to ammonia, were significantly higher in SJSW samples than in the other three seawater samples (Fig. 7A). The abundances of NarB (0.53% ± 0.14%) and NirA (0.38% ± 0.05%) were significantly higher in the SJSW samples than in the other three seawater samples (not significant between SJSW and SRSW for NirA), indicating higher assimilatory reduction of nitrate to ammonia (Fig. 7A). The denitrification pathway from nitrite to nitric oxide (NirK, 0.75% ± 0.17%), nitrous oxide (NorBC, 0.61% ± 0.15%), and nitrogen (NosZ, 0.49% ± 0.14%) were also higher in the SJSW samples. The nitrogen fixation (NirDKH and AnfG, 0.23% ± 0.06%) in the SJSW samples was only significantly higher than in the NSW samples. The first step of nitrification (AmoABC) in the SJSW (0.03% ± 0.02%), SCSW (0.02% ± 0.01%), and SRSW (0.02% ± 0.03%) was higher than in the NSW samples (0.01% ± 0.01%), but no significant difference was observed in the subsequent processes. No significant enrichment of the anammox pathway was observed in the seawater samples. For the rhizosphere sediment samples, no consistent trend was found in any of the six nitrogen metabolism pathways (Fig. 7B). The dissimilatory reduction of nitrate to nitrite was lower in the SJRS (NarGHI, 4.25% ± 0.25%) and SCRS (NapA, 3.66% ± 0.41%) samples, whereas the assimilatory reduction of nitrate to nitrite was higher in the SJRS (NarB, 0.30% ± 0.12%) and SCRS (NR, 0.05% ± 0.03%) samples. Interestingly, the nitrogen fixation pathway in the SJRS was lower than the other three rhizosphere sediment samples, which was opposite to the seawater samples (Fig. 7B).

The sulfur metabolism pathways were also analyzed to investigate the difference between those four zones. Generally, the assimilatory sulfate reduction and dimethylsulfoniopropionate (DMSP) degradation proteins were higher in the seawater samples, while the dissimilatory sulfate reduction and oxidation genes were higher in the rhizosphere sediment samples (Fig. S14). The assimilatory reduction of sulfate was similar in the seawater samples between those four zones (Fig. S15). However, the assimilatory reduction of sulfate from phosphoadenosine phosphosulfate (PAPS) to sulfite (CysH) and sulfide (CysJI) in the SJRS (1.91% ± 0.17% and 2.09% ± 0.36%) and SCRS (1.61% ± 0.20% and 1.97% ± 0.10%) samples was higher than in the NRS (1.19% ± 0.13% and 1.19% ± 0.28%) and SRRS (0.98% ± 0.11% and 0.83% ± 0.12%) samples (Fig. S15). The dissimilatory reduction and oxidation processes (AprAB, between APS and sulfite) in the NSW (1.85% ± 0.31%) and SJSW (1.94% ± 0.43%) samples were higher than the other two seawater samples, but dissimilatory reduction and oxidation between sulfite and sulfide were higher in the SRSW samples (DsrAB, 0.33% ± 0.22%, Fig. S15A). In the rhizosphere sediment samples, the dissimilatory reduction and oxidation between adenylyl sulfate (APS), sulfite, and sulfide (AprAB and DsrAB) in SJRS (4.01% ± 0.48% and 2.44% ± 0.35%) samples was lower compared with that in the other three rhizosphere sediment samples (Fig. S15B). Only SoxCD genes were detected in the thiosulfate to sulfate oxidation (SOX) system, and these genes were higher in the SJSW (1.58% ± 0.11%) and SCSW (1.79% ± 0.10%) samples but were lower in the SJRS (0.73% ± 0.12%) and SCRS (0.56% ± 0.03%) samples when compared with the other two seawater and rhizosphere sediment samples. The dimethylsulfoniopropionate (DMSP) demethylation pathway (mediated by the DmdA enzyme) showed high activity in the NSW (2.35% ± 0.05%) and SJSW (2.43% ± 0.30%) samples in addition to the SJRS samples (0.40% ± 0.12%), while the cleavage pathway (the DddL enzyme) was lower both in the SJSW (0.11% ± 0.05%) and SJRS (0.06% ± 0.02%) samples when compared with the corresponding seawater and rhizosphere sediment samples (Fig. S14).

### Antibiotics resistance

Considering the addition of antibiotics to mariculture, antibiotic resistance in the seawater and rhizosphere sediment environment may have changed in the SJ and SC zones. We analyzed the differences and enrichment of ARGs in seawater and rhizosphere sediment samples from those four zones. The composition of the ARGs in the seawater and rhizosphere sediment samples from the SJ and SC zones was similar, in that the multidrug resistance genes were the main ARGs (37.28%~42.09% and 45.06%~48.43%) followed by MLS (11.96%~13.84% and 9.33%~10.67%), glycopeptide (10.04%~10.80% and 10.11%~12.46%), and tetracycline (10.41%~11.23% and 8.71%~10.36%) resistance genes (Fig. S16). The LEfSe analysis of ARGs showed that the NSW (six ARGs) and SCSW (six ARGs) samples have more enriched ARGs than the SJSW (three ARGs) and SRSW (four ARGs) samples. However, this pattern was opposite in the rhizosphere sediment samples which SJRS (10 ARGs) and SRRS (5 ARGs) samples had more enriched ARGs (Fig. S17). Triclosan resistance genes were enriched both in the NSW (1.00% ± 0.10%) and NRS (1.56% ± 0.06%) samples, while bicyclomycin resistance genes were enriched in the SJSW (0.23% ± 0.01%) and SJRS (0.13% ± 0.01%) samples. The pleuromutilin resistance genes were enriched both in the SCSW (3.37% ± 0.27%) and SCRS (2.04% ± 0.09%) samples, and fluoroquinolone resistance genes were enriched both in the SRSW (2.32% ± 0.13%) and SRRS (2.35% ± 0.12%) samples. Tetracycline (11.23% ± 0.39% and 10.36% ± 0.13%), sulfonamide (2.20% ± 0.17% and 0.78% ± 0.06%), and diaminopyrimidine (1.28% ± 0.10% and 0.52% ± 0.03%) resistance genes were usually enriched in the mariculture environment (SCSW and SJRS samples, Fig. S17).

## Discussion

The interactions between seagrass and microbial communities are essential for seagrass adaptation and the homeostasis of seagrass meadows through metabolic exchanges and biogeochemical transformations [4, 5]. Microorganisms in seawater and rhizosphere sediment benefit the seagrass regarding nitrogen and sulfur cycling [4, 64], which may be affected by environmental factors. We investigated the impacts of mariculture on the environmental microbiome surrounding *Z. marina* in seawater and rhizosphere sediments, focusing on community composition, metabolic pathways, and functional genes.

### Rhizosphere sediment has higher levels of bacterial diversity, internal relations, and nitrogen metabolic pathways than seawater

The most striking result was the significant difference in community diversity and richness between seawater and rhizosphere sediment samples in all four zones. Especially for the bacterial community, the rhizosphere sediment samples possessed higher diversity (Shannon index) and richness (OTU number and Chao 1 index) as shown in Fig. S2. These results agree with Shang et al., who found a higher Shannon index for the bacterial community in sediment rather than in seawater but not for the fungal community [65]. This finding may have been because the rhizosphere sediments contain more nutrients than the corresponding seawaters and could provide adequate substrates for seagrass growth [66]. The NMDS results showed that the bacterial, eukaryotic, and fungal communities in seawaters were separated from the rhizosphere sediments, revealing the distinct difference between these two biotopes (Fig. S8). Previous studies addressing the microbial community also revealed the significant difference between seawaters and sediments in the coastal and ocean environments [67, 68]. The Mantel test results revealed that the top three abundant families/phyla of bacterial, eukaryotic, and fungal communities were significantly correlated with diameters in the rhizosphere sediment samples (Fig. 4), which may be due to the effect of nutrient access, transport, and respiration in this habitat [69, 70]. Zai et al. found that root diameter differentially affected rhizospheric bacterial communities and implied a close relationship between the bacterial microbiome, root functions, and soil properties, and this finding confirmed our results that diameters of the rhizosphere were correlated with bacterial, eukaryotic, and fungal communities [71]. PO_4_^3−^ concentration may be the key factor influencing these top three abundant families/phyla, which has been proven in previous studies [72–77]. Besides, in seawater samples, the NH_4_^+^ concentration was another important factor that affected the top three abundant families of bacterial communities, and the majority of these taxa were involved in nitrogen metabolism [78, 79].

No significant differences in the phylum of *Proteobacteria* in addition to the eukaryotic taxa were found in our study, a result that was different from Shang et al. who found a significantly higher level of *Proteobacteria* in rhizosphere sediments [65]. These differences may result from the various environmental conditions in which our two seagrass meadows (SJ and SC zones) were influenced by mariculture. The region-specific characteristics of eukaryotic and fungal communities in both seawater and rhizosphere sediment may reveal their susceptibility to environmental impacts in seagrass meadows. For example, *Trebouxiophyceae* in a previous study was related to clay and silt [80], but no such enrichment of this taxon was found in the zones with higher clay and silt level (SRRS samples, 11.47% and 65.46%, respectively). Chlorophyta (mainly the *Trebouxiophyceae* and *Ulvophyceae*) were found to be related to PO_4_^3−^ and NH_4_^+^ concentrations (Fig. 4B, E). This finding may suggest their demands for the phosphate and ammonium nutrients in these natural and mariculture environments, which had been reported to influence the health of *Trebouxiophyceae* and *Ulvophyceae*, such as the fatty acid proportions [75, 81], the photosynthesis rates [82], and the morphology of algae [83, 84]. SRRSW samples may be the main contributors to these correlations considering the highest phosphate concentration. Significant correlations were observed between Arthropoda (mainly Hexanauplia, the crustaceans) and PO_4_^3−^ concentrations. Calcium phosphate was crucial for the formation of mineralized chitinous exoskeleton [85], and its lowest concentration in the SJSW may suggest the limitation for the Hexanauplia growth in the SJSW samples (Fig. 1B).

The significantly enhanced KEGG pathways in seawaters were mainly the global and overview maps of metabolism, but the rhizosphere sediments possessed diverse enhanced pathways such as carbon metabolism, two-component system, quorum sensing, and oxidative phosphorylation (Fig. S11C). Bacteria were found to possess the highest contributions to most of the KEGG metabolic pathways, especially the secondary metabolites, microbial metabolism in diverse environments, biosynthesis of amino acids, and carbon metabolism, while the contributions of viruses were the lowest especially in rhizosphere sediments. It is reported that seagrass-related bacteria could increase the nitrogen availability for uptake by seagrass, mineralizing amino acids, thus enhancing the growth and productivity of seagrass meadows [86]. These higher bacterial contributors may suggest their essential roles in maintaining healthy seagrass growth in our study. Seagrass rhizomes and roots could release significant amounts of dissolved organic carbon and stimulate microbial activity, including sulfate reduction in the rhizosphere in turn [24]. However, viruses played important roles in nucleotide metabolism, replication and repair, which was even higher than bacteria (Fig. S12). Some studies have revealed that viruses could cause alterations in the nucleotide metabolism pathways and DNA repair of hosts [87–89], which confirmed the genetic information related role of viral components in the environment surrounding seagrass. These findings suggest that bacteria may be the vital component in the microbiome of the seagrass surrounding environment, and viruses may affect genetic information processing.

The nitrogen metabolic proteins, especially those in the dissimilatory nitrate reduction, denitrification, and nitrogen fixation pathways, were higher in rhizosphere sediment samples than in seawater samples (Fig. S13), indicating that the rhizosphere was a hotspot of nitrogen metabolism. These findings are consistent with previous knowledge that higher fixation levels [90, 91], denitrification [92, 93], and dissimilatory nitrate reduction [94] were found in the rhizosphere or bulk sediment of seagrass meadows than in the overlying seawater. Denitrification could reduce NO_3_^−^ and NO_2_^−^ to N_2_ thus removing reactive nitrogen from a biological system, and dissimilatory nitrate reduction reduces NO_3_^−^ to NH_4_^+^ and contributes to the nitrogen retention of seagrass [95]. The competition between denitrification and dissimilatory nitrate reduction could regulate nitrogen elimination/retention dynamics in the seagrass holobiont in our study. This finding was confirmed by Aoki et al. who revealed the importance of denitrification and dissimilatory nitrate reduction to ammonium formation in the seagrass root zone using a push-pull incubation method [94]. Assimilatory sulfate reduction and DMSP degradation were higher in seawaters, while the dissimilatory sulfate reduction and oxidation were higher in the rhizosphere sediments (Fig. S14). It is known that an increase in sulfite concentration could harm seagrass health and result in dieback [96]. The variation in the sulfate reduction in the seawater and rhizosphere sediment of the *Z. marina* seagrass would impact its homeostasis.

### *S. Japonica* culture enhances bacterial internal relations, nitrogen metabolism, and sulfate reduction levels

The richness and diversity of the bacterial community in SJSW samples were higher than in the NSW and SRSW samples, indicating that the culture of *S. japonica* may cause an increase in the complexity of the bacterial community in the seawater environment (Figs. S2A and 2D). This finding was confirmed by the bacterial co-occurrence network that the SJSW samples had more edges and positive correlations (Fig. 3). Studies have shown that kelp cultures could enhance coastal biogeochemical cycles by maintaining bacterioplankton richness [97], and dissolved organic carbon released by *S. japonica* may be the key matter that enhances the stability of the bacterial community [37, 98]. *Psychromonas* was enriched in the SJSW samples, which had previously been found in the epiphytic bacteria of *S. japonica* [99]. It is an important primary degrader of both proteins and lipids and could produce alginate lyases [100, 101]. Approximately 50% of brown seaweed cell walls and intracellular material are alginate; thus, the enriched *Psychromonas* in the SJSW samples may indicate the degradation demands of *S. Japonica*. Members of *Flavobacteriaceae* and *Rhodobacteraceae* were enriched in SJRS samples, which were mainly detected in the diseased *S. japonica* [102]. This enrichment may be related to the degradation of disease kelp and subsequently decomposition in the SJ zone. *Desulfosarcinaceae* (*Desulfobacteraceae*) and *Desulfobulbaceae* are known as the dominant sulfate-reducing bacteria [4], and these enriched families revealed a potentially higher level of sulfate-reducing process in the SJRS samples. Besides, Liu et al. found an increasing sulfate level in kelp blanching water [103], and the high SO_4_^2−^ (9.11 ± 4.49 mmol/kg) concentrations in SJRS samples may be due to dead kelp degradation. Thus, these sulfate-reducing bacteria cause an increase and then help reduce SO_4_^2−^.

Nitrogen metabolism was significantly enriched in the SJSW samples based on the LEfSe results (Fig. 6A). After an in-depth analysis, we found higher dissimilatory and assimilatory nitrate reduction processes in the SJSW samples when compared with the other three kinds of seawaters surrounding seagrass. Nitrogen fixation and the first step of nitrification were also higher in the SJSW samples when compared with the NSW samples (Fig. 7A). These findings revealed that the nitrogen metabolism in the seawater surrounding seagrass in the SJ zone was higher than in the other three zones. The cultivation of *S. japonica* needs a sufficient nitrate supply which limited their productivity [104, 105], and sufficient nitrate enhances the nitrogen metabolism in SJ zone. Wang et al. found that nitrate reductase activity and nitrate absorptivity were found to be fairly high in the *S. japonica* sporophyte [106], which support the higher nitrogen metabolism in the SJ zone. No consistently significant differences were found for the nitrogen metabolism between SJRS and the other three kinds of rhizosphere sediment samples, indicating that the impact of *S. japonica* culture on seagrass rhizosphere was less than that of the seawater. Sandy marine sediments could provide a significant source of nitrogen to the water column in which it was difficult to preserve nutrients [107]; thus, the seawater may have higher nitrogen metabolism rates than in the sediment environment considering the highest sand proportions in SJRS samples (71.87–95.82%). Furthermore, the highest NO_2_^−^ and NO_3_^−^ concentrations were observed in the SJSW and SJRS samples, which also confirmed the presence of active nitrogen metabolism in the SJ zone (Tables S3 and S4). The CCA results suggested that the bacterial, eukaryotic, and fungal communities in the SJSW and SJRS samples were both influenced by NO_3_^−^ and NO_2_^−^ concentrations, especially the bacterial and fungal communities in seawater. This finding further confirmed the nitrogen metabolism results above (Figs. S9 and S10). Many of the metabolic pathways for *S. japonica* substrates, including the metabolism of amino acids, lipids, and xenobiotics, were enriched in the SJRS samples, and these enrichments may due to the demand of the *S. japonica* residues degradation process [108]. The abundance of dissimilatory nitrate reduction proteins was lower in the SCSW and SCRS samples compared to the NSW and NRS samples. On the contrary, the assimilatory nitrate reduction proteins were higher in the SCSW and SCRS samples than in the NSW and NRS samples. Zheng et al. found nitrate and ammonium uptake, which helps remove nitrogen from the aquaculture systems, in the sediment of the sea cucumber culture pond [109]. Nitrogen in the sea cucumber culture is enriched due to animal feces, extra feed, and fertilizers [110]. Thus, the excess nitrate in the sea cucumber culture may be removed from the seagrass meadows via assimilatory nitrate reduction in the SC zone.

The assimilatory nitrate reduction from PAPS to sulfide was generally higher in the SJSW, SJRS, and SCRS samples, which indicates that the *S. japonica* and sea cucumber culture may have brought sulfide pressure to the seagrass. Furthermore, *Sulfurovum* could oxidize sulfide and thiosulfate to sulfate [111, 112], and the enrichment of this genus in the SCRS samples may have helped mitigate the sulfide pressure of seagrass in the SC zone. Choi et al. revealed that sulfur metabolism was a key driver of refractory dissolved organic carbon production during the biodegradation of *S. japonica* [113], and the higher level of sulfate reduction and in the SJ zone may have been due to the requirements of *S. japonica* degradation, which may bring sulfite pressure for seagrass in the meantime. The level of sulfite pressure may increase in the *S. japonica* and sea cucumber culture (SJ and SC) zones as revealed by an increase in SO_4_^2−^ concentration, assimilatory sulfate reduction, and sulfate-reducing related bacteria. Thus, artificial breeding scales should be under control and supervision to preserve seagrass meadows from degradation risk.

Considering the enriched microorganisms in all these natural and maricultural zones, it was found that most of them were related to sulfate and nitrogen metabolism, which were shown to assist seagrass in nitrite removal and nitrogen fixation [114]. Members of *Sulfitobacter* (enriched in NSW) are specializing in sulfite oxidation, and these marine bacteria are abundant in the seagrass tissues and could protect algae such as *Emiliania huxleyi* from pathogen infection [115]. *Sulfurovum* sp. (enriched in SCRS) could use oxygen and nitrate as electron acceptors and gain energy by oxidizing reduced sulfur compounds through the sulfur-oxidizing (Sox) pathway [116]; thus, this enrichment may be a response to sea cucumber culture for maintaining the seagrass health in our study. MAGs of Desulfobacterales and Desulfuromonadales, mainly the sulfur-reducing bacteria, were obtained from our metagenome data, and these bacteria were commonly associated with anoxic, sulfide-rich seagrass rhizosphere [117]. The annotation of these MAGs also revealed the sulfur-reducing ability in these bacteria (DsrC, DsrD), and maybe potential seagrass probiotics (Table S6). These associations between seagrass and sulfide-oxidizing bacteria could alleviate toxic sulfate accumulation, produce higher biomass and form more complex rhizome structures [118]. Besides, some other enriched bacteria, such as *Psychromonas* (enriched in SJSW) [119] and Rhodobacteraceae (enriched in SJRS) [120], are capable of nitrite reduction, nitrate assimilation and/or nitrogen fixation, which may aid *Z. marina* in maintaining health under kelp culture pressure. MAGs of Rhodobacteraceae were also obtained from the metagenomic data, and these bacteria had potential nitrogen fixation ability with NifU annotated. Further, MAGs in the family Akkermansiaceae contained genes for dissimilatory nitrate reduction, which was consistent with Weigel’s work [121], and these bacteria might potentially promote the growth of *Z. marina* considering its nitrate reduction ability. Although there was no study revealed the effects of *Lentimonas* and *Polaribacter* to the growth of seagrass, genes involving in fucoidan degradation [122] (alpha-l-fucosidase) were observed in MAGs of these two genera. Considering that fucoidan is mainly derived from kelp, bacteria of *Lentimonas* and *Polaribacter* could provide nutrients for seagrass growth through degrading dead kelp, being seagrass probiotics.

Different amounts of antibiotics have been used in the mariculture and are released into the marine and terrestrial environments [123]. Although the predominant ARGs in these four zones were similar, the ARGs against bicyclomycin, pleuromutilin, tetracycline, sulfonamide, and diaminopyrimidine were significantly enriched in the mariculture zones (SJ and SC zones, Fig. S16). Bicyclomycin, sulfonamides, and tetracyclines have been widely used in mariculture to treat bacterial and protozoan infections [124, 125]. ARG enrichment indicates the impact of antibiotic addition to the seagrass surrounding environment, which may enhance their ARG levels compared to the natural sea. Effective supervision measures should be taken to reduce the addition of antibiotics in mariculture to protect seagrass meadow ecosystems.

## Conclusions

This study analyzed the impacts of mariculture on the environmental microbiome surrounding the seagrass *Z. marina*, focusing on the bacterial, eukaryotic, and fungal components in the composition, diversity, metabolism, and responses to the mariculture environment. We found significant differences in the composition, richness, diversity, and internal relations of the bacterial community between the seawater and rhizosphere sediments surrounding *Z. marina*, while the eukaryotic and fungal communities were less significant. More complex bacterial and fungal co-occurrence networks were found in the seawater and rhizosphere sediments of the SJ and SC zones. The SJSW samples had higher levels of dissimilatory and assimilatory nitrate reduction, denitrification, and nitrogen fixation processes than the other three zones. Proteins related to assimilatory sulfate reduction were higher in the rhizosphere sediments of the SJ zone. Tetracycline, sulfonamide, and diaminopyrimidine resistance genes were enriched in the mariculture zones. Our study showed the impact of mariculture on the microbiome of seagrass *Z. marina* and could enhance the understanding of the mariculture impacts on seagrass meadow ecosystems. These novel insights may help evoke the emphasis on the human productive activity impacts on natural ecosystems.

### Supplementary Information

**Additional file 1: **Supplementary materials. Results of bacterial, eukaryotic, and fungal community structure, biological functions and metabolism in the seawater and rhizosphere sediment of *Z. marina* from natural and mariculture zones. **Fig. S1.** The four sampling zones. **Fig. S2.** The difference of α diversity in seawater (A, B and C) and rhizosphere sediment (D, E and F) samples from the N, SJ, SC and SR zones. **Fig. S3.** The composition of the metagenomic communities at domain level. **Fig. S4.** The composition of bacterial, eukaryotic, and fungal communities in seawater (A, B and C) and rhizosphere sediment (D, E and F) samples from the N, SJ, SC and SR zones at genus level. **Fig. S5.** The phylogenomic tree and numbers of MAGs assembled from seawater and rhizosphere sediment samples in the N, SJ, SC and SR zones. **Fig. S6.** The co-occurrence network of eukaryotic community in seawater (A, B, C and D) and rhizosphere sediment (E, F, G and H) samples from the N, SJ, SC and SR zones. **Fig. S7.** The co-occurrence network of fungal communities in seawater (A, B, C and D) and rhizosphere sediment (E, F, G and H) samples from the N, SJ, SC and SR zones. **Fig. S8.** The nonmetric multidimensional scaling (NMDS) analysis of bacterial (A), eukaryotic (B) and fungal (C) communities at ASV level based on the Bray-Curtis. **Fig. S9.** The canonical correspondence analysis (CCA) of bacterial, eukaryotic, and fungal communities in seawater (A, B and C) and rhizosphere sediment (D, E and F) samples from the N, SJ, SC and SR zones at ASV level. **Fig. S10.** The variation partition analysis (VPA) of bacterial, eukaryotic, and fungal communities in seawater (A, B and C) and rhizosphere sediment (D, E and F) samples from the N, SJ, SC and SR zones at ASV level. **Fig. S11.** The Kyoto Encyclopedia of Genes and Genomes (KEGG) functions of the metagenome in seawater (A) and rhizosphere sediment (B) samples and their difference (C). **Fig. S12.** The Kyoto Encyclopedia of Genes and Genomes (KEGG) functions in bacterial, eukaryotic, archaeal and viral communities in seawater and rhizosphere sediment samples from the N, SJ, SC and SR zones. **Fig. S13.** The nitrogen metabolism in seawater and rhizosphere sediment samples from the N, SJ, SC and SR zones. **Fig. S14.** The sulfur metabolism in seawater and rhizosphere sediment samples from the N, SJ, SC and SR zones. **Fig. S15.** The sulfur metabolism in seawater (A) and rhizosphere sediment (B) samples from the N, SJ, SC and SR zones. **Fig. S16.** The antibiotic class of antibiotic resistance genes (ARGs) in seawater (A) and rhizosphere sediment (B) samples from the N, SJ, SC and SR zones. **Fig. S17.** The LEfSe analysis for the antibiotic class of antibiotic resistance genes (ARGs) in seawater (A) and rhizosphere sediment (B) samples from the N, SJ, SC and SR zones. **Table S1.** Primers and amplification conditions for the high-throughput sequencing of bacteria, eukaryota and fungi. **Table S2.** The qualities of metagenomic assemblies. **Table S3.** The environmental parameters of the seawater samples from the N, SJ, SC and SR zones. **Table S4.** The environmental parameters of the rhizosphere sediment samples from the N, SJ, SC and SR zones. **Table S5.** The ASVs numbers, Chao 1, Shannon and Coverage indices.

## Data Availability

The raw reads of 16S rRNA, 18S rRNA, ITS genes, and metagenomic sequencing were deposited into the Sequence Read Archive of the National Center for Biotechnology Information (NCBI) with the accession number SRR24965703 to SRR24965734, SRR24967515 to SRR24967546, SRR24967574 to SRR24967605, and SRR25010643 to SRR25010674, respectively, under BioProject PRJNA985394. All the MAGs were deposited into the NCBI Genomes as biosample SAMN38508403 to SAMN38508511 under bioproject PRJNA985394.

## Abbreviations

ITS: Internal transcribed spacer
MAGs: Metagenome-assembled genomes
PAPS: 3′-Phosphoadenosine-5′-phosphosulfate
APS: Adenosine phosphosulfate
DMSP: Dimethylsulfoniopropionate
ARGs: Antibiotic resistance genes
