# Supplementary material for: Novel insights into the rhizosphere and seawater microbiome of Zostera marina in diverse mariculture zones
Source: Microbiome. 2024 Feb 14;12:27. doi: 10.1186/s40168-024-01759-3 (PMC10865565; doi:10.1186/s40168-024-01759-3)
Supplement: Supplementary file 2 — Additional file 1: Supplementary materials. Results of bacterial, eukaryotic, and fungal community structure, biological functions and metabolism in the seawater and rhizosphere sediment of Z. marina from natural and mariculture zones. Fig. S1. The four sampling zones. Fig. S2. The difference of α diversity in seawater (A, B and C) and rhizosphere sediment (D, E and F) samples from the N, SJ, SC and SR zones. Fig. S3. The composition of the metagenomic communities at domain level. Fig. S4. The composition of bacterial, eukaryotic, and fungal communities in seawater (A, B and C) and rhizosphere sediment (D, E and F) samples from the N, SJ, SC and SR zones at genus level. Fig. S5. The phylogenomic tree and numbers of MAGs assembled from seawater and rhizosphere sediment samples in the N, SJ, SC and SR zones. Fig. S6. The co-occurrence network of eukaryotic community in seawater (A, B, C and D) and rhizosphere sediment (E, F, G and H) samples from the N, SJ, SC and SR zones. Fig. S7. The co-occurrence network of fungal communities in seawater (A, B, C and D) and rhizosphere sediment (E, F, G and H) samples from the N, SJ, SC and SR zones. Fig. S8. The nonmetric multidimensional scaling (NMDS) analysis of bacterial (A), eukaryotic (B) and fungal (C) communities at ASV level based on the Bray-Curtis. Fig. S9. The canonical correspondence analysis (CCA) of bacterial, eukaryotic, and fungal communities in seawater (A, B and C) and rhizosphere sediment (D, E and F) samples from the N, SJ, SC and SR zones at ASV level. Fig. S10. The variation partition analysis (VPA) of bacterial, eukaryotic, and fungal communities in seawater (A, B and C) and rhizosphere sediment (D, E and F) samples from the N, SJ, SC and SR zones at ASV level. Fig. S11. The Kyoto Encyclopedia of Genes and Genomes (KEGG) functions of the metagenome in seawater (A) and rhizosphere sediment (B) samples and their difference (C). Fig. S12. The Kyoto Encyclopedia of Genes and Genomes (KEGG) functions in bacteri [file 40168_2024_1759_MOESM1_ESM.docx]

# Novel insights into the rhizosphere and seawater microbiome of *Zostera marina* in diverse mariculture zones

**Hao Sun^1^, Tianyu Wang^1^, Shuai Liu^1^, Xiaoyu Tang^2^, Jie Sun^3^, Xuerui Liu^1^, Ye Zhao^1^, Pingping Shen^1^, Yanying Zhang^1^***

^1^ School of Ocean, Yantai University, Yantai, 264005, China

^2^ CAS Key Laboratory of Tropical Marine Bio-resources and Ecology, South China Sea Institute of Oceanology, Guangzhou 510301, China

^3^ Fisheries College, Ocean University of China, Qingdao, 266003

***Correspondence:**

Yanying Zhang, Email: zhyanying@ytu.edu.cn

Running title: the microbiome surrounding *Zostera marina*





**Fig. S1 The four sampling zones.** N, natural sea zone. SJ, *Saccharina japonica* culture zone, SC, sea cucumber culture zone. SR, seagrass restoration zone.


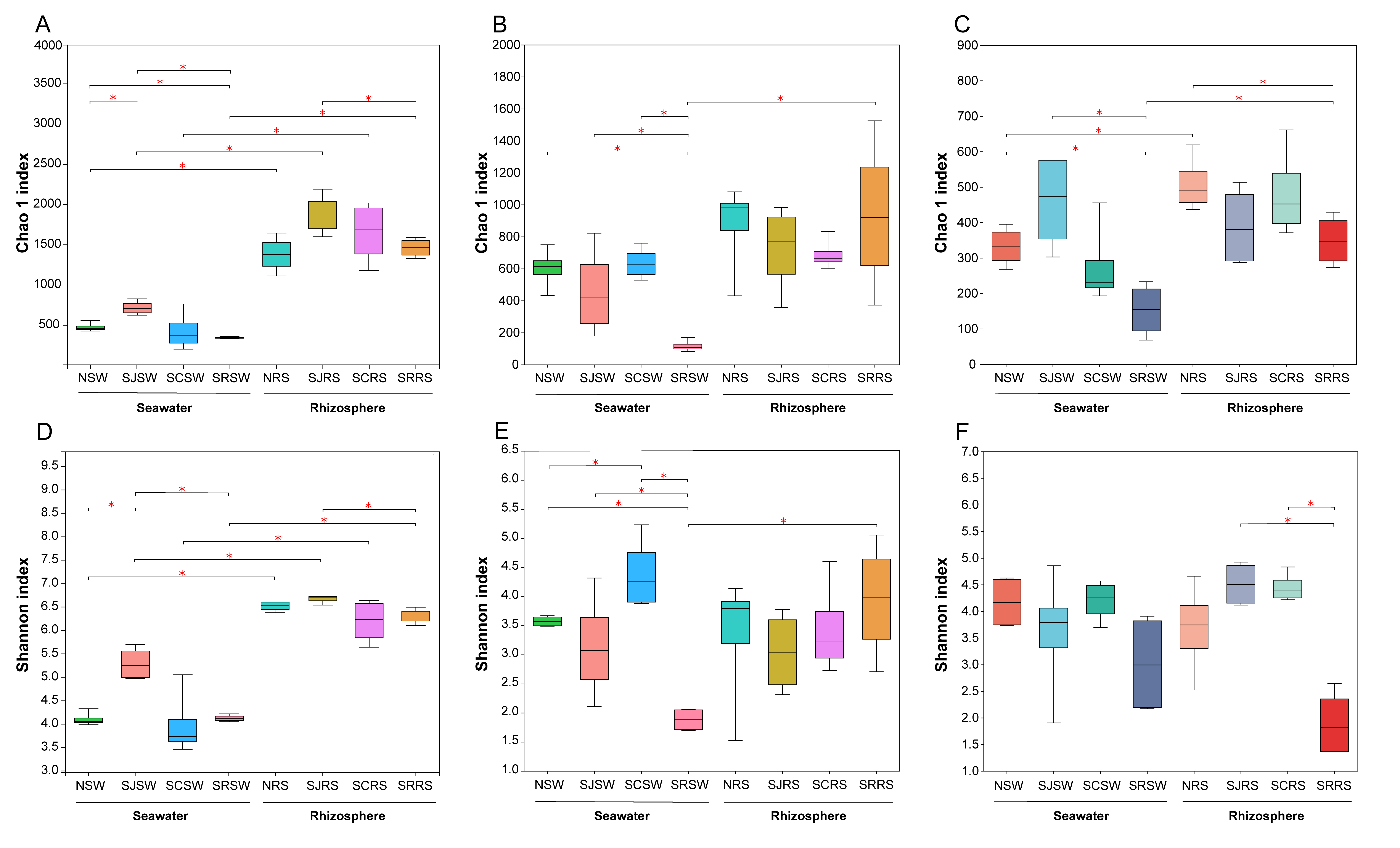


**Fig. S2 The difference of α diversity in seawater (A, B and C) and rhizosphere sediment (D, E and F) samples from the N, SJ, SC and SR zones.** NSW, SJSW, SCSW and SRSW, the sewater samples of N, SJ, SC and SR zones. NRS, SJRS, SCRS and SRRS, the rhizosphere sediment samples of N, SJ, SC and SR zones. Wilcoxon rank-sum test is used to determine the difference between the N, SJ, SC and SR zones. *P* < 0.05 is marked with “*”.


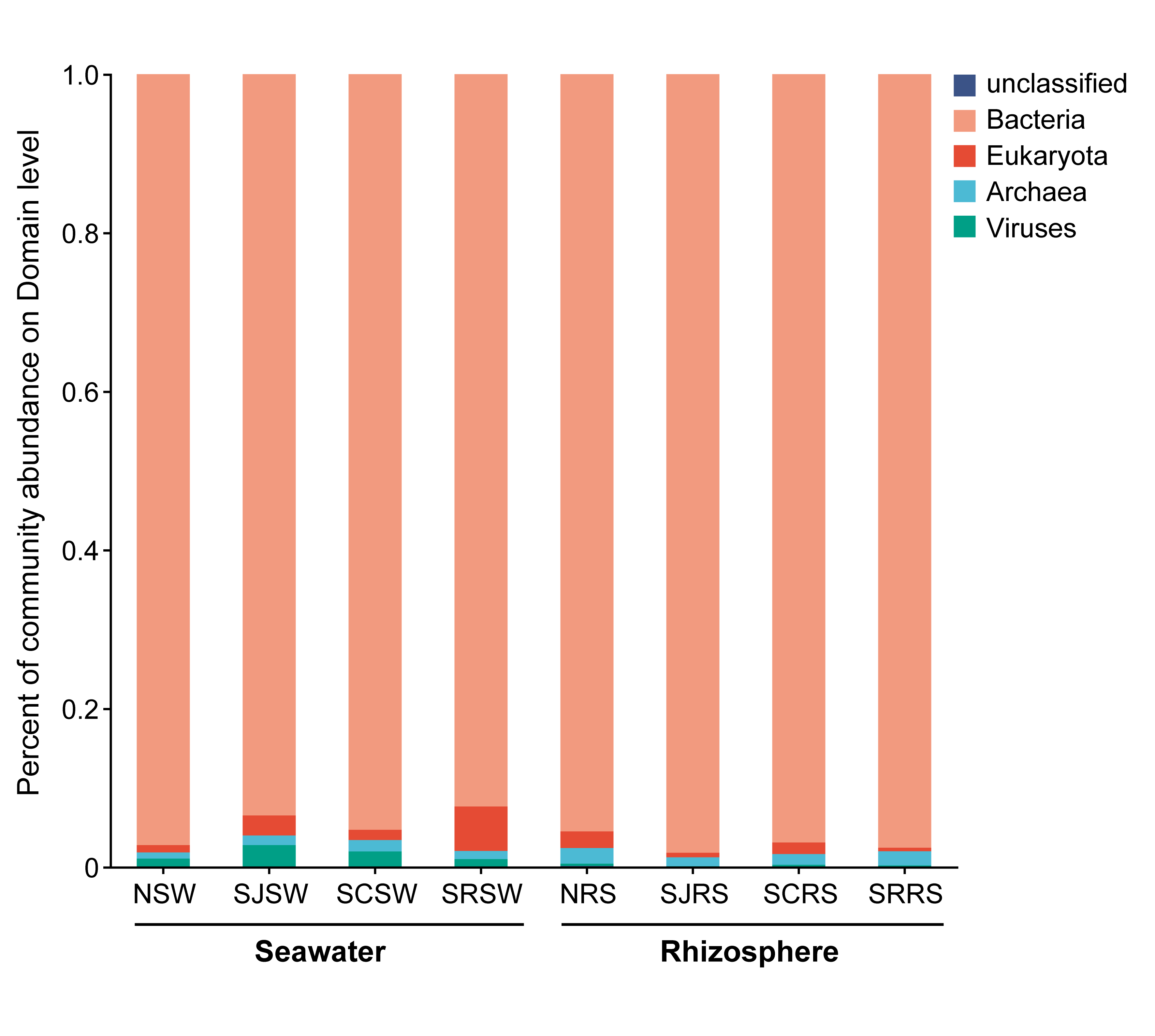


**Fig. S3 The composition of the metagenomic communities at domain level.** Figure was constructed from metagenome assemblies using R program with “ggplot2” package according to the taxonomy assignment based on NR database. The reads per kilobase million (RPKM) is used to calculated the relative abundance. NSW, SJSW, SCSW and SRSW, the sewater samples of N, SJ, SC and SR zones. NRS, SJRS, SCRS and SRRS, the rhizosphere sediment samples of N, SJ, SC and SR zones.


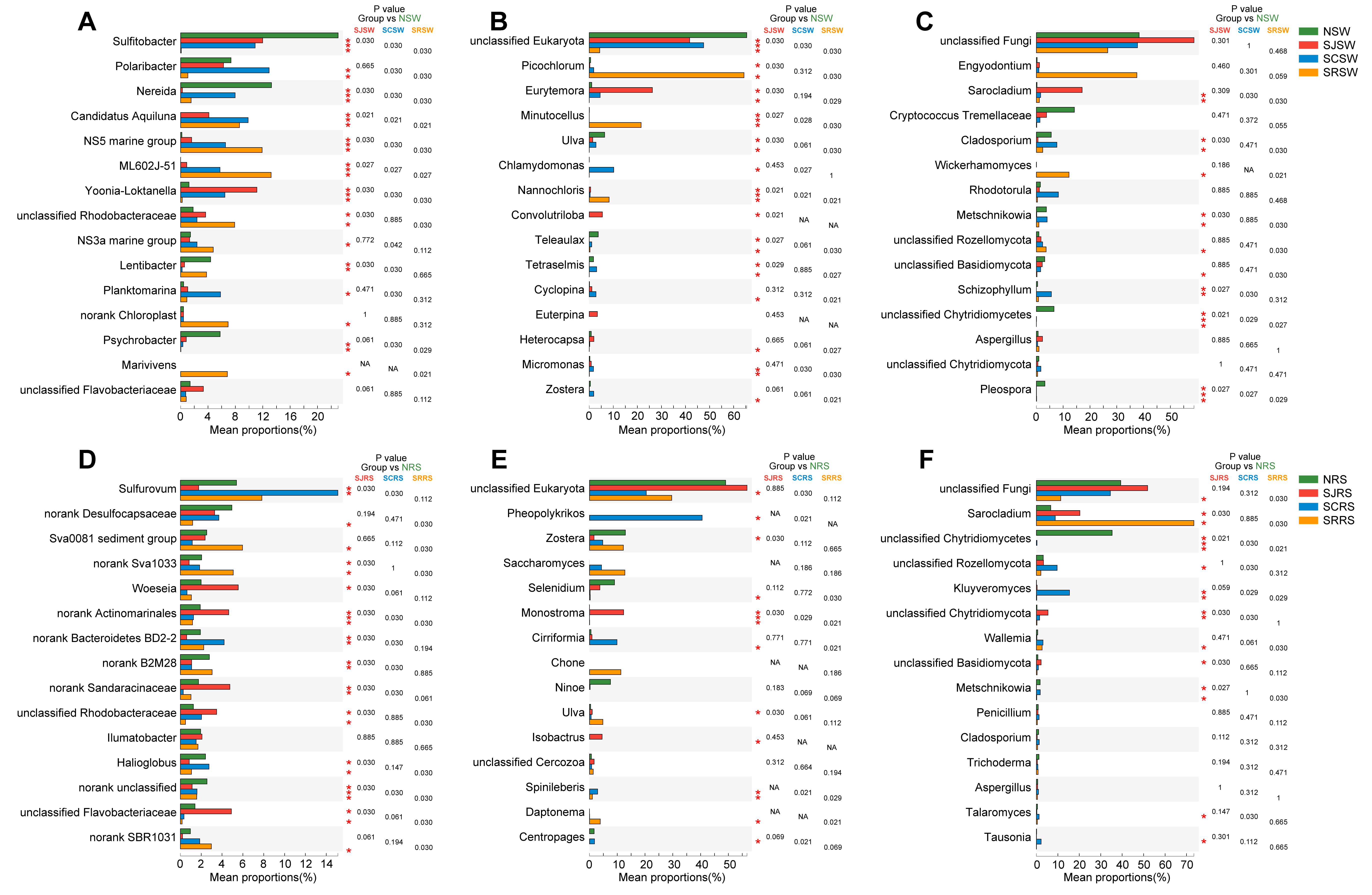


**Fig. S4 The composition of bacterial, eukaryotic and fungal communities in seawater (A, B and C) and rhizosphere sediment (D, E and F) samples from the N, SJ, SC and SR zones at genus level.** NSW, SJSW, SCSW and SRSW, the sewater samples of N, SJ, SC and SR zones. NRS, SJRS, SCRS and SRRS, the rhizosphere sediment samples of N, SJ, SC and SR zones. Wilcoxon rank-sum test is used to determine the difference between the N, SJ, SC and SR zones. *P* < 0.05 is marked with “*”.


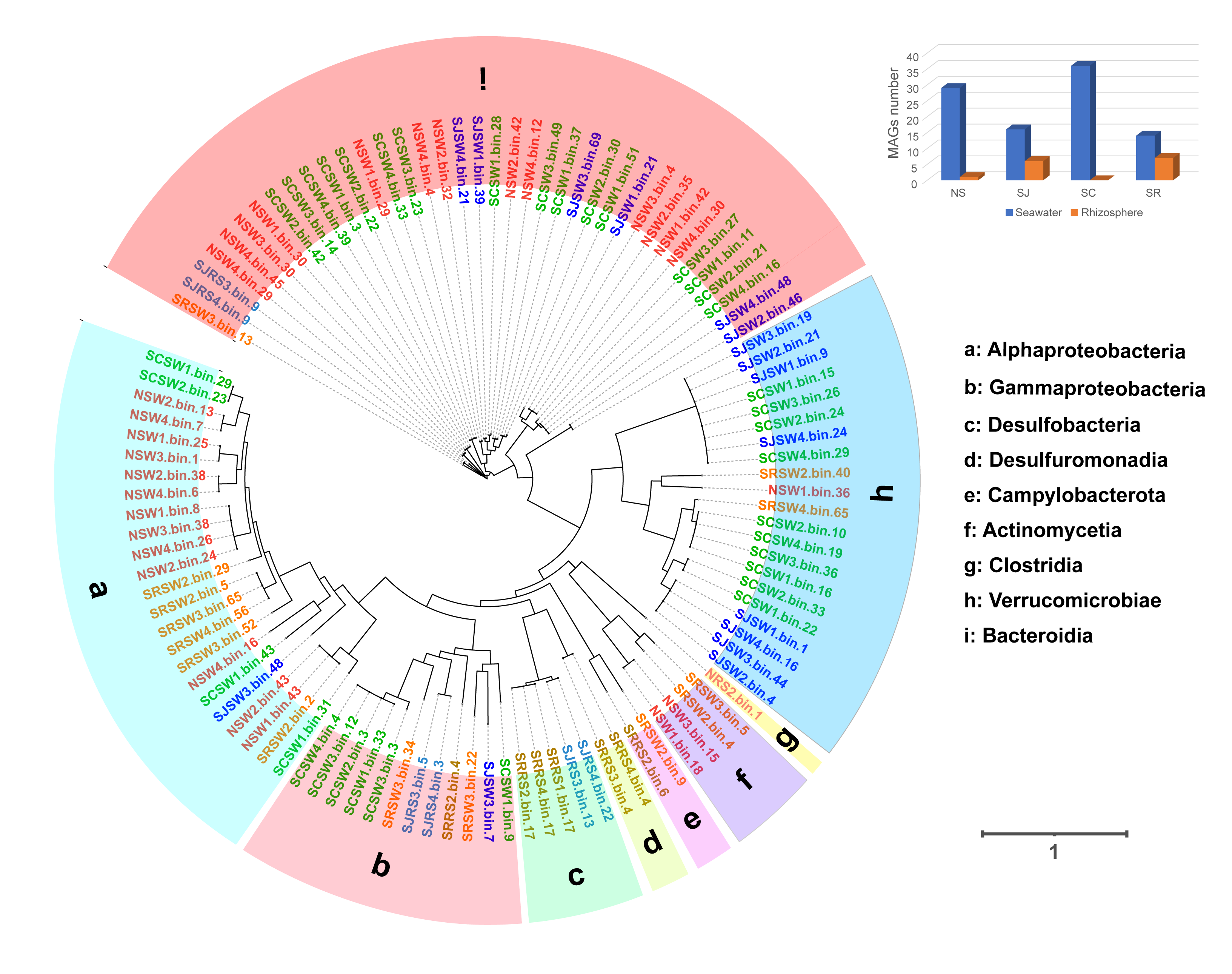


**Fig. S5 The phylogenomic tree and numbers of MAGs assembled from** **seawater and rhizosphere sediment samples in the N, SJ, SC and SR zones**. 120 single-copy core genes were used to construct the phylogenomic tree. NSW, SJSW, SCSW and SRSW, the sewater samples of N, SJ, SC and SR zones. NRS, SJRS, SCRS and SRRS, the rhizosphere sediment samples of N, SJ, SC and SR zones.





**Fig. S6 The co-occurrence network of eukaryotic community in seawater (A, B, C and D) and rhizosphere sediment (E, F, G and H) samples from the N, SJ, SC and SR zones.** ASVs of the top 100 abundance in each sample are selected. NSW, SJSW, SCSW and SRSW, the sewater samples of N, SJ, SC and SR zones. NRS, SJRS, SCRS and SRRS, the rhizosphere sediment samples of N, SJ, SC and SR zones. The colors of spots indicate different eukaryotic phyla, and lines between spots indicate the correlations between ASVs. Red lines, positive correlations. Blue lines, negative correlations.





**Fig. S7** **The** **co-occurrence network of fungal communities in seawater (A, B, C and D) and rhizosphere sediment (E, F, G and H) samples from the N, SJ, SC and SR zones.** ASVs of the top 100 abundance in each sample are selected. NSW, SJSW, SCSW and SRSW, the sewater samples of N, SJ, SC and SR zones. NRS, SJRS, SCRS and SRRS, the rhizosphere sediment samples of N, SJ, SC and SR zones. The colors of spots indicate different fungal phyla, and lines between spots indicate the correlations between ASVs. Red lines, positive correlations. Blue lines, negative correlations.


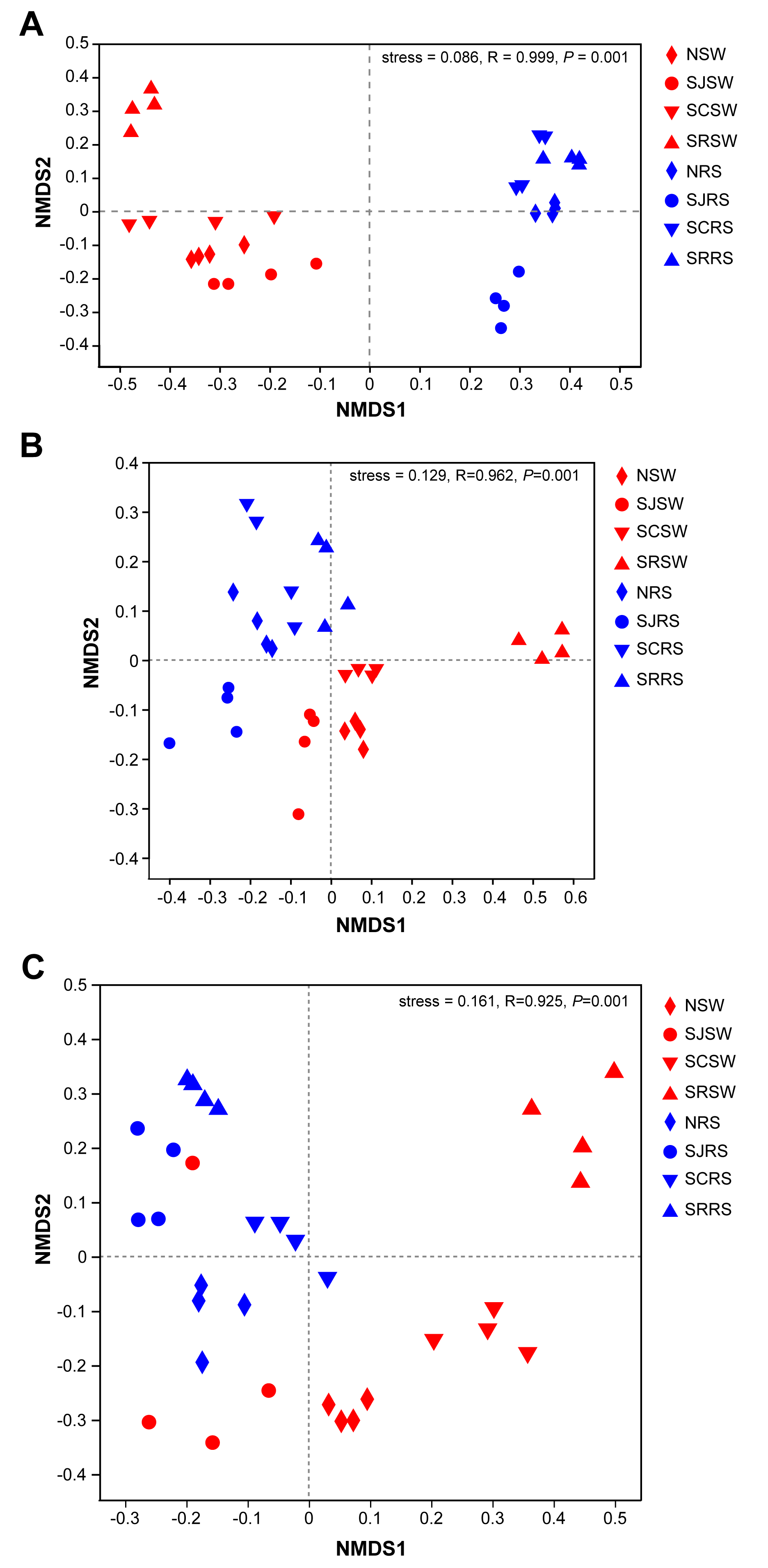


**Fig. S8 The nonmetric multidimensional scaling (NMDS) analysis of bacterial (A), eukaryotic (B) and fungal (C) communities at ASV level based on the Bary-Curtis.** NSW, SJSW, SCSW and SRSW, the sewater samples of N, SJ, SC and SR zones. NRS, SJRS, SCRS and SRRS, the rhizosphere sediment samples of N, SJ, SC and SR zones. Red and blue sports indicate seawater and rhizosphere sediment samples.


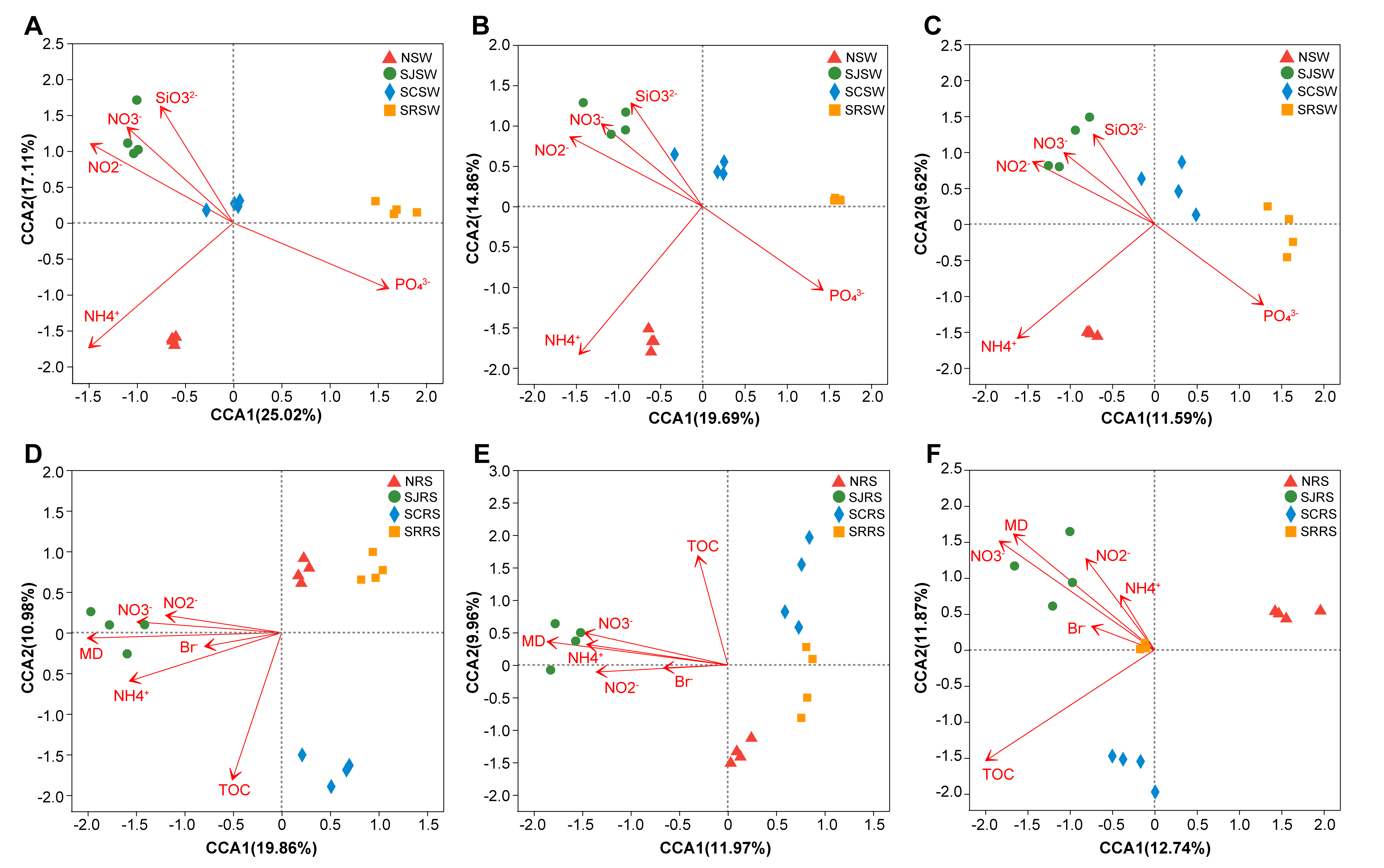


**Fig. S9 The** **canonical correspondence analysis (CCA) of bacterial, eukaryotic and fungal communities in seawater (A, B and C) and rhizosphere sediment (D, E and F) samples from the N, SJ, SC and SR zones at ASV level.** NSW, SJSW, SCSW and SRSW, the sewater samples of N, SJ, SC and SR zones. NRS, SJRS, SCRS and SRRS, the rhizosphere sediment samples of N, SJ, SC and SR zones.


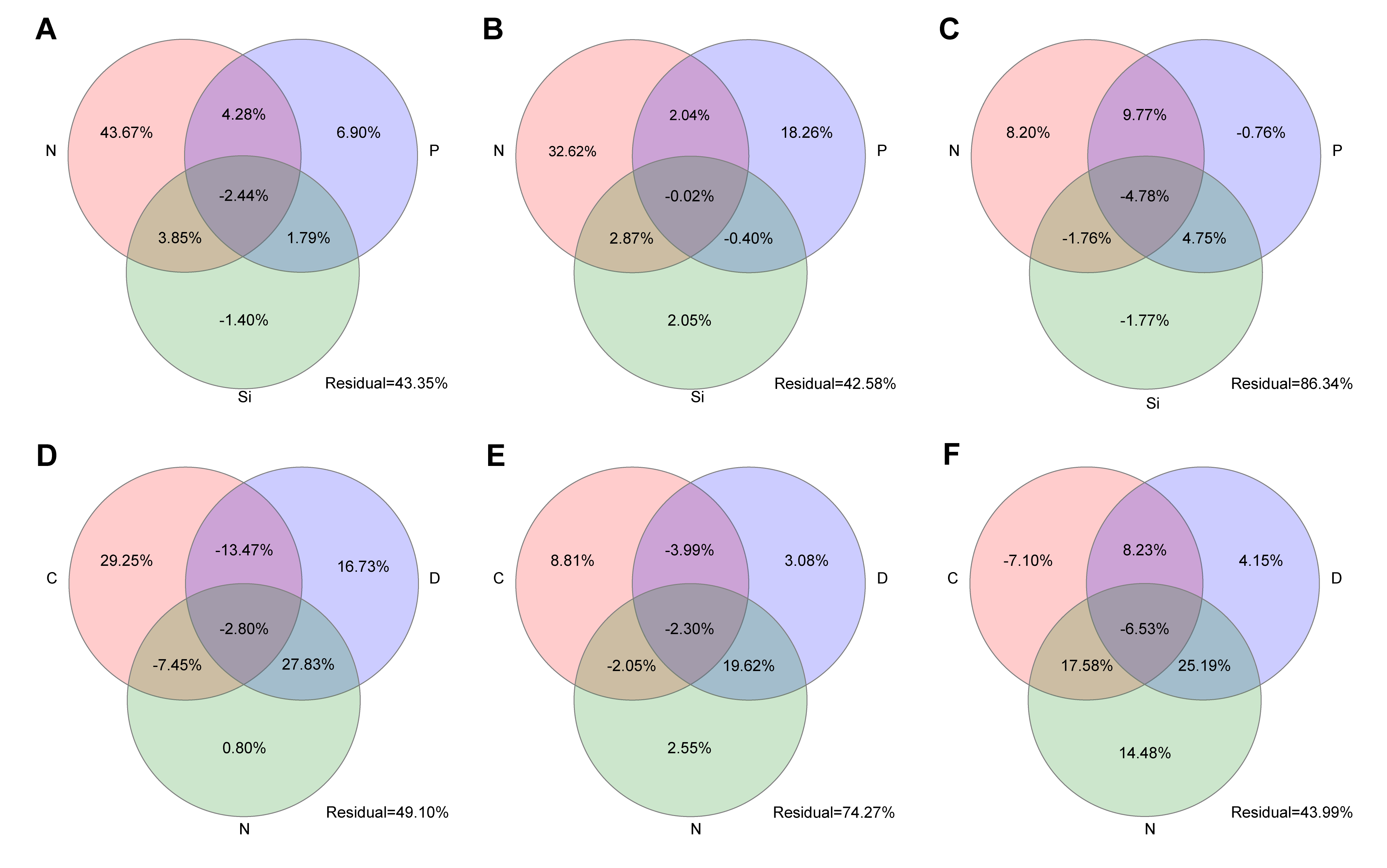


**Fig. S10 The variation partition analysis (VPA) of bacterial, eukaryotic and fungal communities in seawater (A, B and C) and rhizosphere sediment (D, E and F) samples from the N, SJ, SC and SR zones at ASV level.** C includes TC and TOC. N includes NO_3_^-^, NO_2_^-^ and NH_4_^+^ in seawater samples while NO_3_^-^, NO_2_^-^, NH_4_^+^ and TON in rhizosphere sediment samples. P includes PO_4_^3-^. Si includes SiO_3_^2-^. D includes MD and D50.


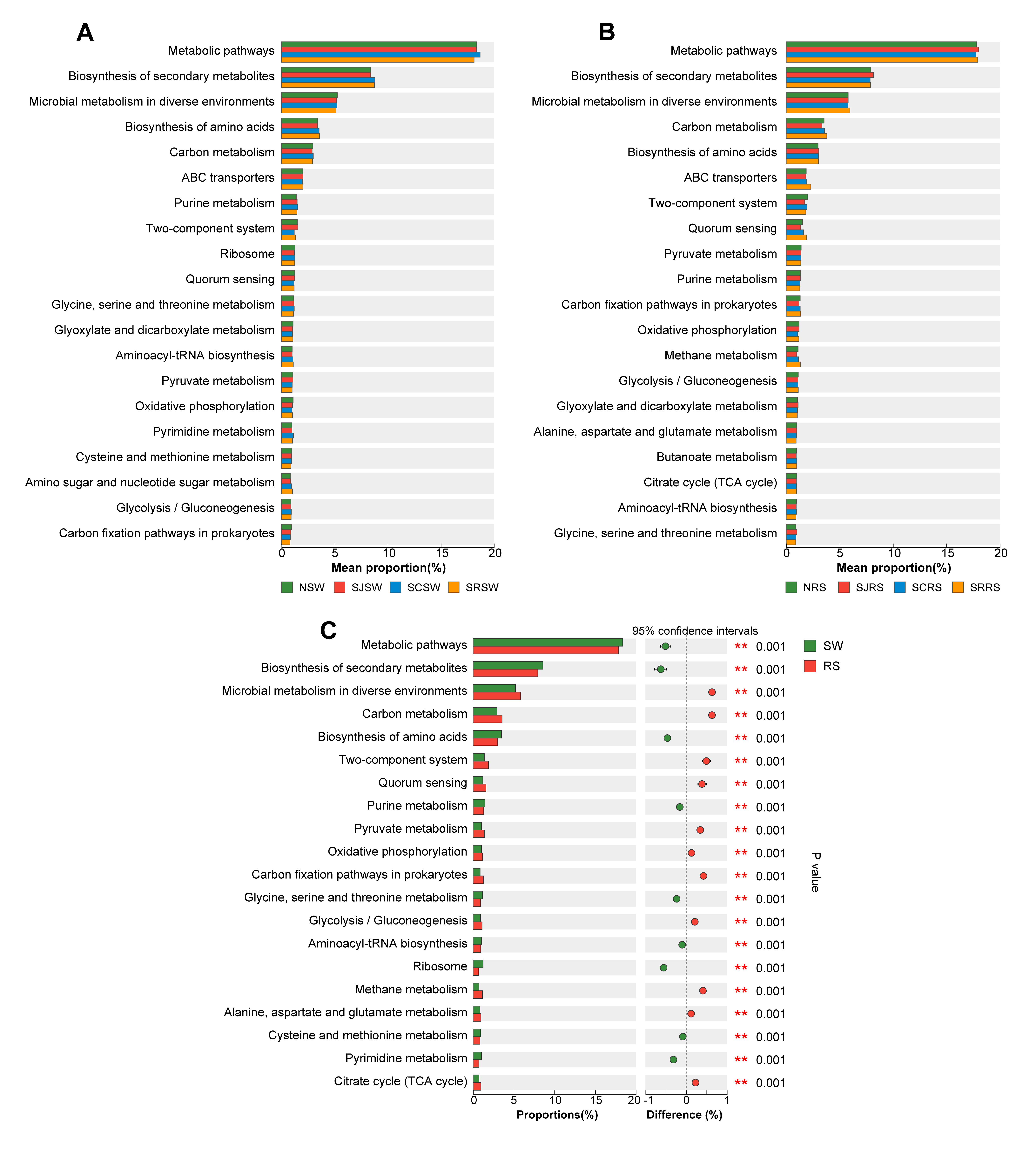


**Fig. S11 The Kyoto Encyclopedia of Genes and Genomes (KEGG) functions of the metagenome in seawater (A) and rhizosphere sediment (B) samples and their difference (C).** Wilcoxon rank-sum test is used to analysis the difference of the KEGG functions between seawater and rhizosphere sediment samples. NSW, SJSW, SCSW and SRSW, the sewater samples of N, SJ, SC and SR zones. NRS, SJRS, SCRS and SRRS, the rhizosphere sediment samples of N, SJ, SC and SR zones. SW, seawater samples. RS, rhizosphere sediment samples.


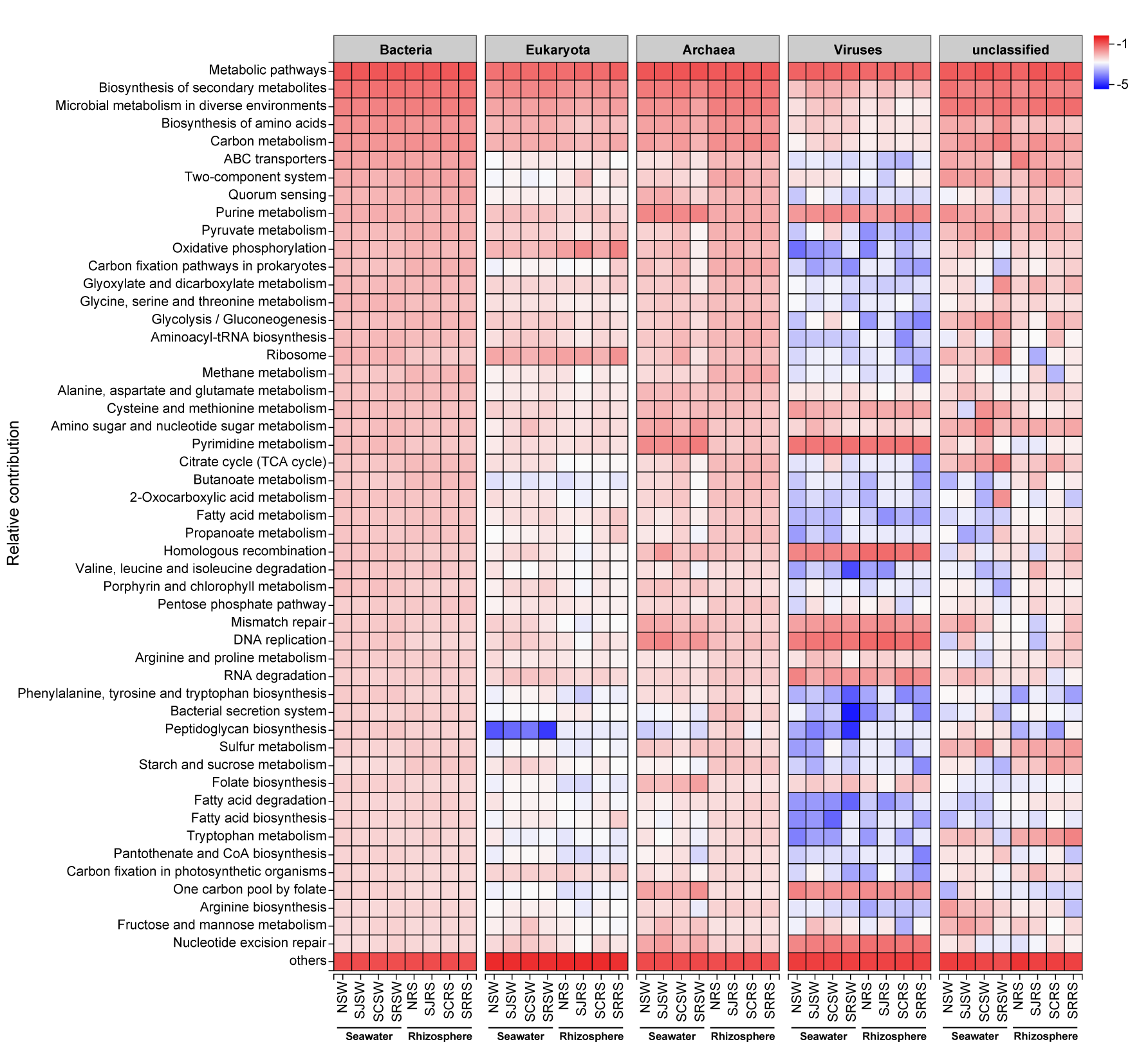


**Fig. S12 The Kyoto Encyclopedia of Genes and Genomes (KEGG) functions in bacterial, eukaryotic, archaeal and viral communities in seawater and rhizosphere sediment samples from the N, SJ, SC and SR zones.** The top 50 KEGG functions are shown. NSW, SJSW, SCSW and SRSW, the sewater samples of N, SJ, SC and SR zones. NRS, SJRS, SCRS and SRRS, the rhizosphere sediment samples of N, SJ, SC and SR zones.


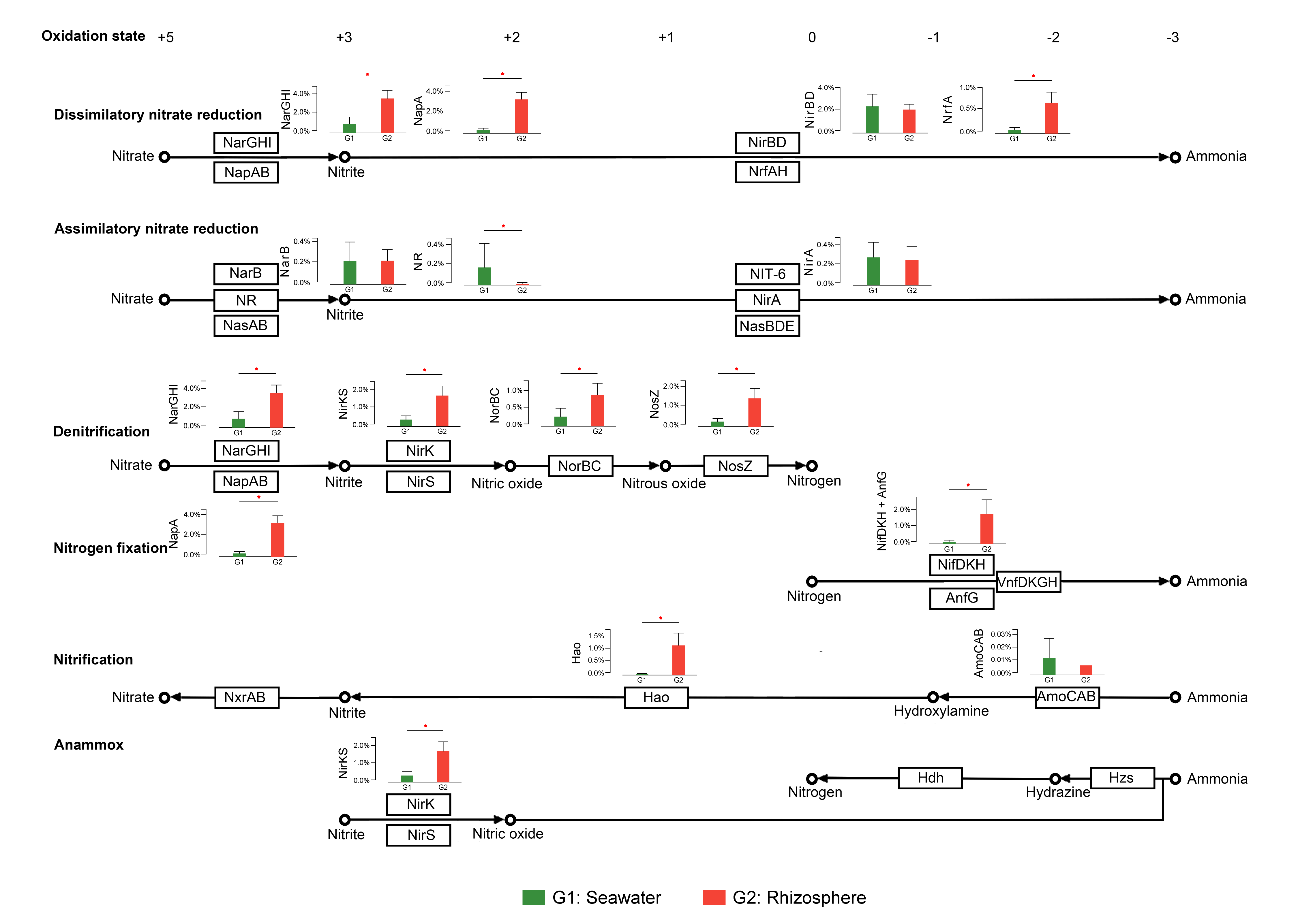


**Fig. S13** **The nitrogen metabolism in seawater and rhizosphere sediment samples from the N, SJ, SC and SR zones.** Wilcoxon rank-sum test is used to analyze the difference of nitrogen metabolic genes between the seawater and rhizosphere sediment samples. *P* < 0.05 is marked with “*”.


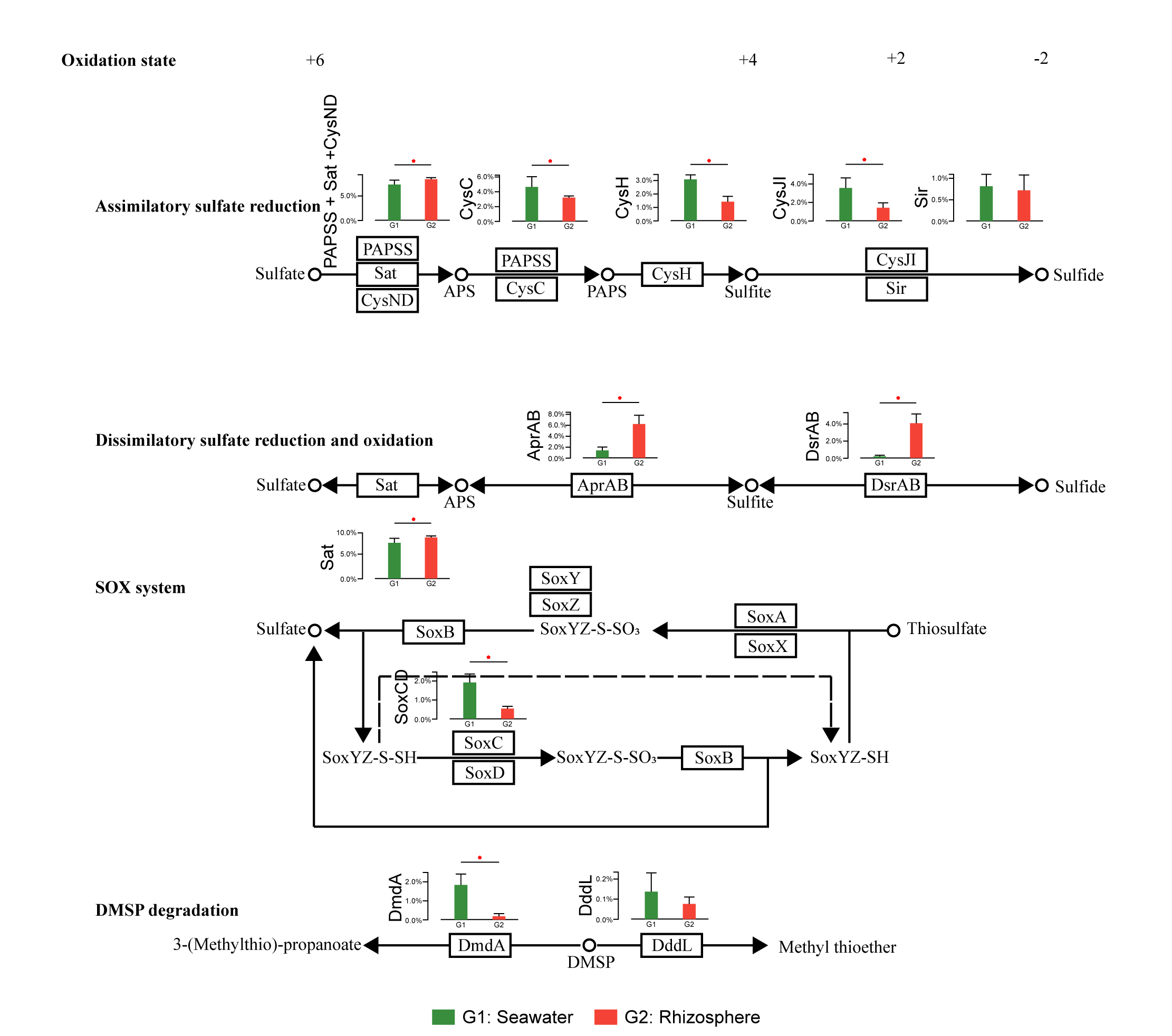


**Fig. S14** **The sulfur metabolism in seawater and rhizosphere sediment samples from the N, SJ, SC and SR zones.** Wilcoxon rank-sum test is used to analyze the difference of sulfur metabolic genes between the seawater and rhizosphere sediment samples. *P* < 0.05 is marked with “*”.


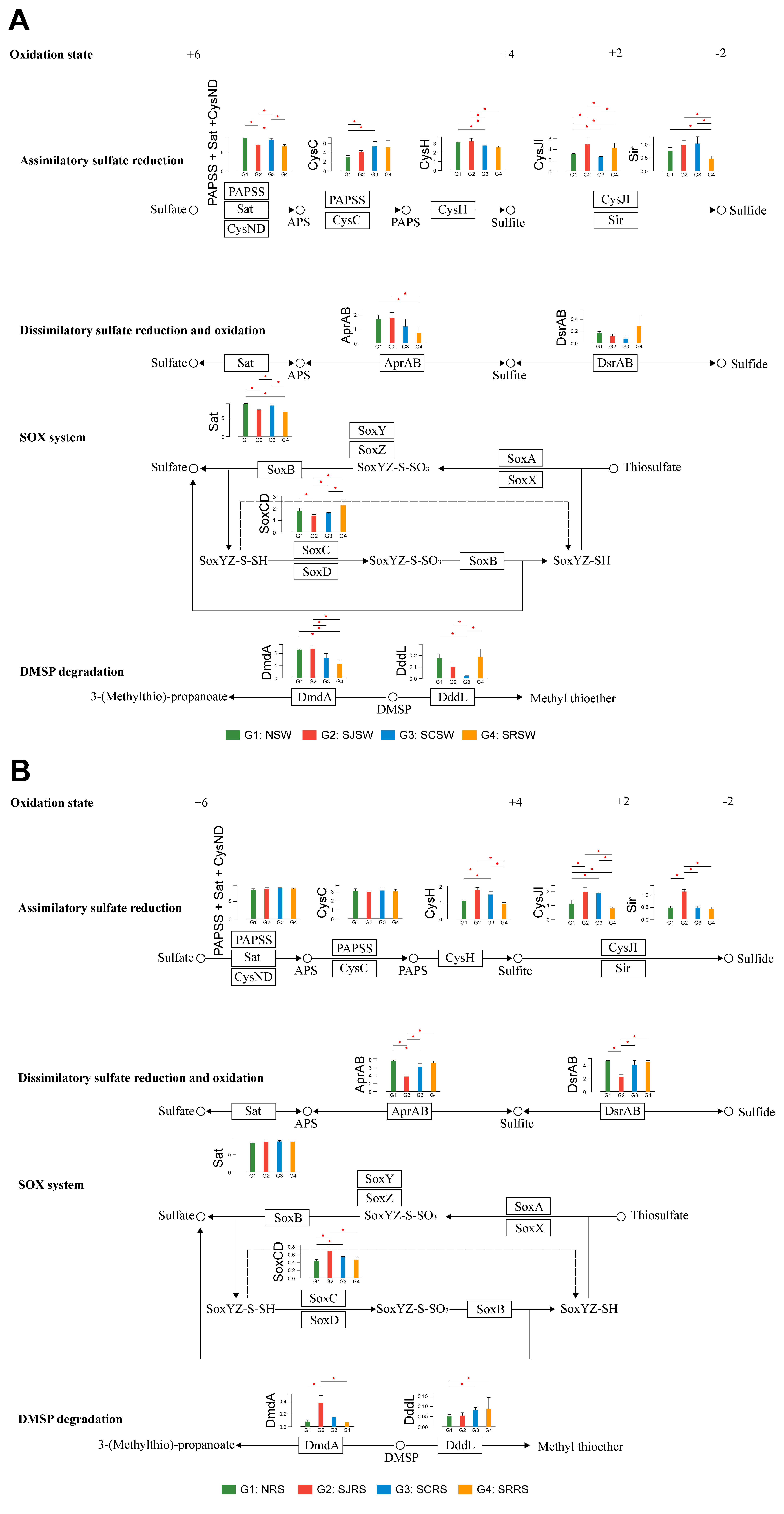


**Fig. S15 The sulfur metabolism in seawater (A) and rhizosphere sediment (B) samples from the N, SJ, SC and SR zones.** NSW, SJSW, SCSW and SRSW, the sewater samples of N, SJ, SC and SR zones. NRS, SJRS, SCRS and SRRS, the rhizosphere sediment samples of N, SJ, SC and SR zones. Wilcoxon rank-sum test is used to analyze the difference of sulfur metabolic genes in the seawater and rhizosphere sediment samples between the N, SJ, SC and SR zones. *P* < 0.05 is marked with “*”.


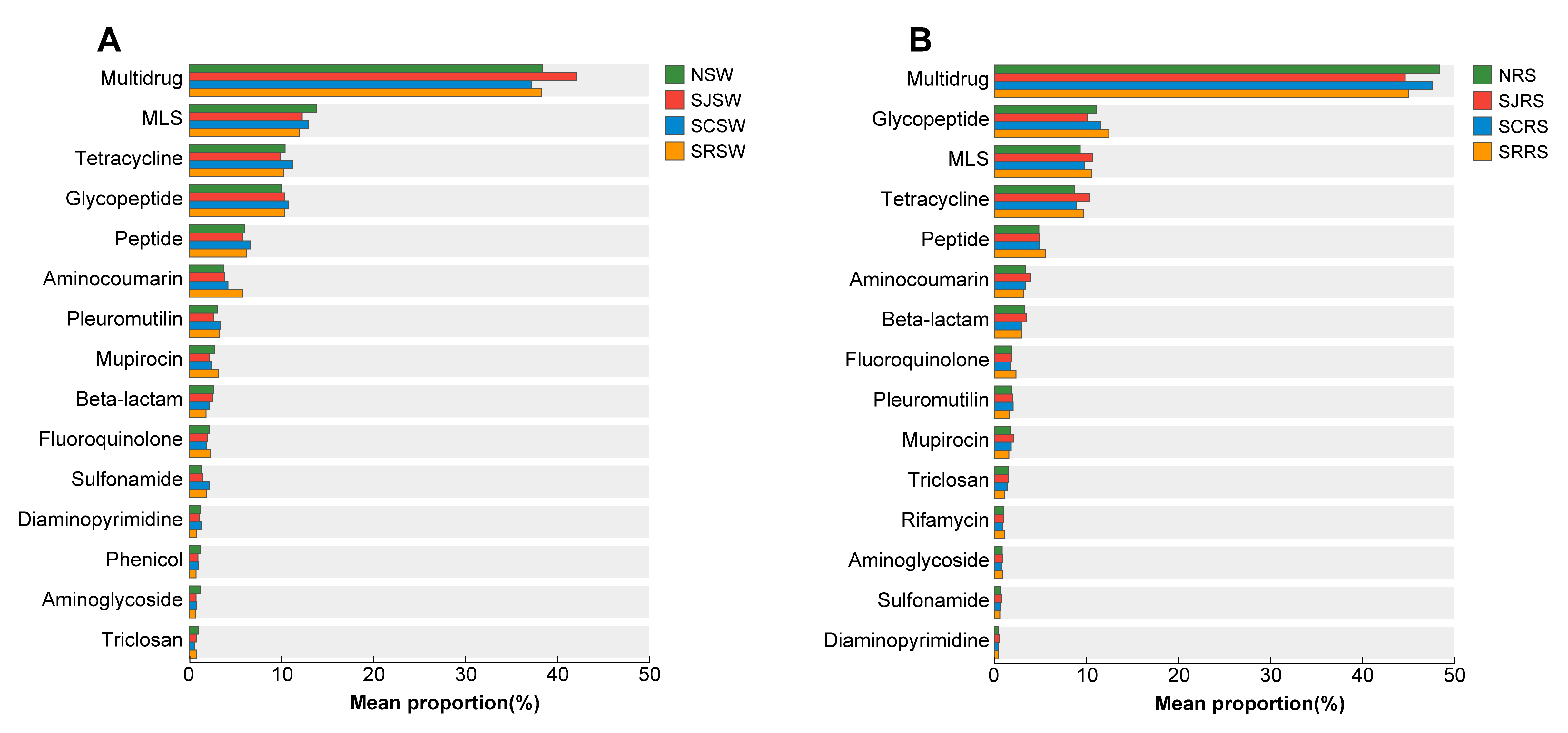


**Fig. S16 The antibiotic class of antibiotic resistance genes (ARGs) in seawater (A) and rhizosphere sediment (B) samples from the N, SJ, SC and SR zones.** NSW, SJSW, SCSW and SRSW, the sewater samples of N, SJ, SC and SR zones. NRS, SJRS, SCRS and SRRS, the rhizosphere sediment samples of N, SJ, SC and SR zones.


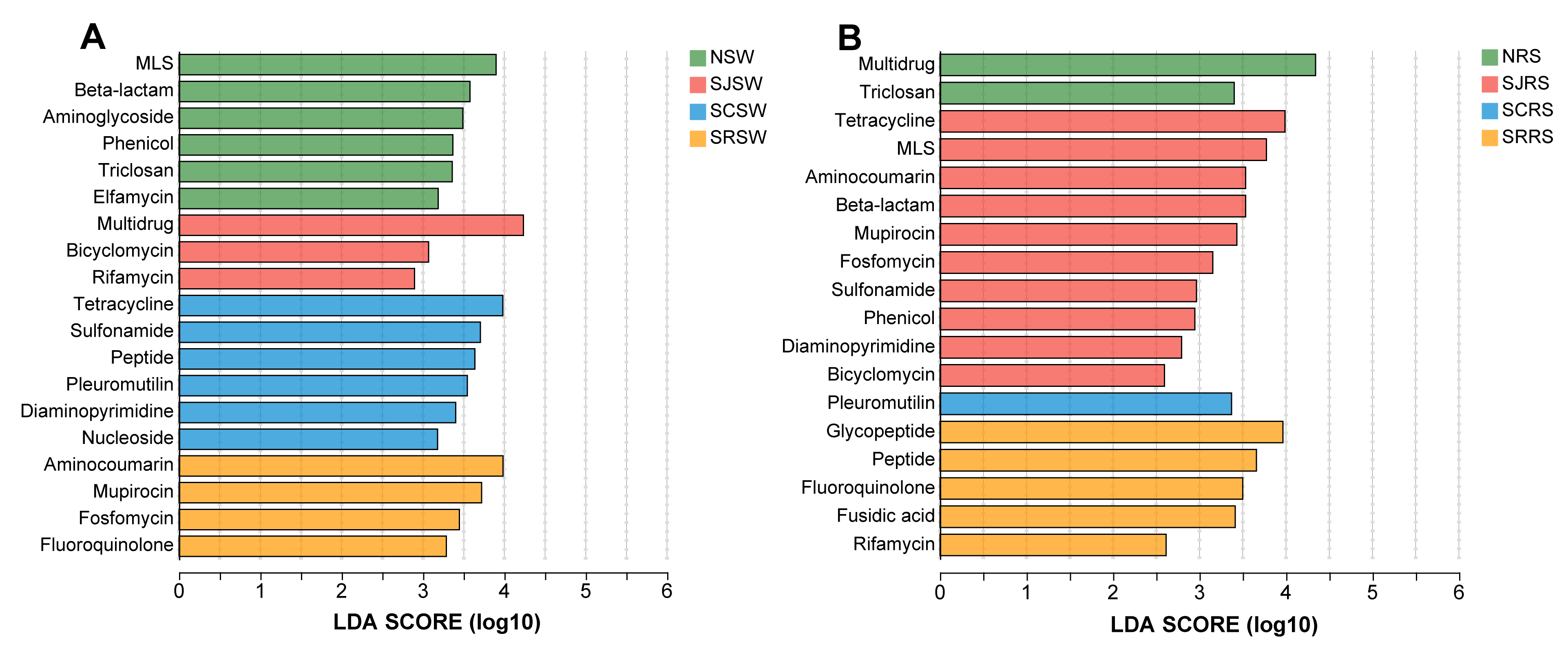


**Fig. S17 The LEfSe analysis for the antibiotic class of antibiotic resistance genes (ARGs) in seawater (A) and rhizosphere sediment (B) samples from the N, SJ, SC and SR zones.** NSW, SJSW, SCSW and SRSW, the sewater samples of N, SJ, SC and SR zones. NRS, SJRS, SCRS and SRRS, the rhizosphere sediment samples of N, SJ, SC and SR zones. Antibiotic class with LDA > 2 is shown.

**Table S1 Primers and amplification conditions for the high-throughput sequencing of bacteria, eukaryota and fungi.**

| Target genes | Primers | Sequences (5’-3’) | Annealing temp (°C) | References |
| --- | --- | --- | --- | --- |
| 16S rRNA | 338F | ACTCCTACGGGAGGCAGCAG | 53 | [1] |
|  | 806R | GGACTACHVGGGTWTCTAAT |  |  |
| 18S rRNA | TAReukFWD1F | CCAGCASCYGCGGTAATTCC | 55 | [2] |
|  | TAReukREV3R | ACTTTCGTTCTTGATYRA |  |  |
| ITS | ITS1F | CTTGGTCATTTAGAGGAAGTAA | 55 | [3] |
|  | ITS2R | GCTGCGTTCTTCATCGATGC |  |  |

**Table S2 The qualities of metagenomic assemblies**

| Samples | Contigs  (≥ 0 bp) | Contigs  (≥ 1000 bp) | Contigs  (≥ 5000 bp) | Contigs  (≥ 10000 bp) | Contig  bases(bp) | N50  (bp) | N90  (bp) | L50 | Max contig  (bp) |
| --- | --- | --- | --- | --- | --- | --- | --- | --- | --- |
| NSW1 | 587861 | 76619 | 5648 | 1536 | 4.36 × 10^8^ | 806 | 355 | 110513 | 535800 |
| NSW2 | 560360 | 74155 | 5564 | 1632 | 4.15 × 10^8^ | 810 | 354 | 104860 | 388712 |
| NSW3 | 654040 | 83847 | 5723 | 1582 | 4.74 × 10^8^ | 776 | 354 | 129845 | 179908 |
| NSW4 | 529098 | 82331 | 5859 | 1763 | 4.16 × 10^8^ | 916 | 358 | 94975 | 201763 |
| SJSW1 | 768417 | 102887 | 5472 | 1391 | 5.50 × 10^8^ | 767 | 357 | 164432 | 133774 |
| SJSW2 | 925773 | 106496 | 4323 | 950 | 6.10 × 10^8^ | 678 | 348 | 219899 | 140122 |
| SJSW3 | 907417 | 130708 | 7746 | 1950 | 6.78 × 10^8^ | 826 | 361 | 179187 | 278908 |
| SJSW4 | 954104 | 110155 | 4590 | 1065 | 6.32 × 10^8^ | 682 | 350 | 227517 | 139577 |
| SCSW1 | 427015 | 70059 | 6369 | 2066 | 3.63 × 10^8^ | 1056 | 367 | 64384 | 290578 |
| SCSW2 | 424340 | 72520 | 5291 | 1673 | 3.54 × 10^8^ | 1028 | 365 | 69372 | 437861 |
| SCSW3 | 657124 | 85821 | 5599 | 1608 | 4.75 × 10^8^ | 780 | 354 | 130975 | 83982 |
| SCSW4 | 582190 | 75372 | 5134 | 1537 | 4.22 × 10^8^ | 783 | 353 | 113212 | 105967 |
| SRSW1 | 544050 | 98166 | 8915 | 2678 | 4.77 × 10^8^ | 1138 | 369 | 81206 | 251478 |
| SRSW2 | 513228 | 97231 | 8976 | 2587 | 4.61 × 10^8^ | 1219 | 372 | 74254 | 234904 |
| SRSW3 | 745093 | 124492 | 7878 | 2036 | 5.94 × 10^8^ | 944 | 365 | 135853 | 161481 |
| SRSW4 | 996434 | 118254 | 5711 | 1245 | 6.61 × 10^8^ | 697 | 345 | 223010 | 161481 |
| NRS1 | 836007 | 41120 | 250 | 13 | 4.19 × 10^8^ | 479 | 326 | 272889 | 23359 |
| NRS2 | 751606 | 69298 | 1923 | 270 | 4.54 × 10^8^ | 615 | 341 | 204559 | 58047 |
| NRS3 | 711840 | 45208 | 797 | 81 | 3.87 × 10^8^ | 541 | 333 | 216683 | 42813 |
| NRS4 | 782813 | 34795 | 328 | 29 | 3.93 × 10^8^ | 492 | 328 | 259813 | 43592 |
| SJRS1 | 814400 | 63268 | 1690 | 201 | 4.70 × 10^8^ | 580 | 338 | 234629 | 26654 |
| SJRS2 | 852581 | 66209 | 1611 | 182 | 4.92 × 10^8^ | 584 | 338 | 245650 | 56220 |
| SJRS3 | 901503 | 85780 | 2376 | 451 | 5.51 × 10^8^ | 614 | 345 | 245158 | 49363 |
| SJRS4 | 1075756 | 116425 | 3477 | 667 | 6.88 × 10^8^ | 656 | 351 | 282614 | 83687 |
| SCRS1 | 1116605 | 87181 | 2289 | 409 | 6.36 × 10^8^ | 559 | 336 | 317648 | 46976 |
| SCRS2 | 587434 | 10751 | 42 | 8 | 2.62 × 10^8^ | 433 | 320 | 218537 | 39845 |
| SCRS3 | 943924 | 104744 | 3449 | 607 | 6.07 × 10^8^ | 663 | 348 | 238667 | 57906 |
| SCRS4 | 760120 | 25951 | 82 | 4 | 3.65 × 10^8^ | 470 | 325 | 264485 | 44734 |
| SRRS1 | 939768 | 71669 | 2873 | 545 | 5.42 × 10^8^ | 555 | 337 | 259127 | 37823 |
| SRRS2 | 761499 | 79231 | 3802 | 723 | 4.90 × 10^8^ | 668 | 345 | 188531 | 48820 |
| SRRS3 | 734266 | 77262 | 2934 | 623 | 4.69 × 10^8^ | 663 | 345 | 186827 | 44169 |
| SRRS4 | 858267 | 89346 | 3333 | 567 | 5.44 × 10^8^ | 649 | 347 | 222115 | 71720 |

**Table S3 The environmental parameters of the seawater samples from the N, SJ, SC and SR zones.**

| Samples | PO_4_^3-^ (μM) | NH_4_^+^ (μM) | NO_2_^-^ (μM) | SiO_3_^2-^ (μM) | NO_3_^-^ (μM) |
| --- | --- | --- | --- | --- | --- |
| NSW1 | 0.424 | 4.748 | 0.183 | 2.541 | 1.285 |
| NSW2 | 0.451 | 4.227 | 0.186 | 2.882 | 1.804 |
| NSW3 | 0.488 | 4.580 | 0.207 | 2.836 | 1.510 |
| NSW4 | 0.454 | 3.710 | 0.188 | 2.875 | 1.901 |
| SJSW1 | 0.188 | 1.823 | 0.243 | 6.430 | 4.212 |
| SJSW2 | 0.166 | 3.020 | 0.226 | 5.921 | 4.391 |
| SJSW3 | 0.350 | 2.856 | 0.683 | 30.990 | 45.435 |
| SJSW4 | 0.344 | 2.634 | 0.676 | 24.287 | 38.810 |
| SCSW1 | 0.326 | 1.537 | 0.129 | 5.784 | 0.723 |
| SCSW2 | 0.391 | 1.774 | 0.107 | 4.696 | 0.509 |
| SCSW3 | 0.225 | 1.921 | 0.109 | 4.586 | 0.531 |
| SCSW4 | 0.221 | 1.733 | 0.174 | 3.672 | 0.514 |
| SRSW1 | 0.474 | 1.621 | 0.093 | 6.046 | 0.105 |
| SRSW2 | 0.676 | 1.687 | 0.098 | 6.220 | 0.107 |
| SRSW3 | 0.334 | 1.423 | 0.067 | 3.682 | 0.083 |
| SRSW4 | 0.779 | 1.602 | 0.112 | 6.309 | 0.202 |

**Table S4 The environmental parameters of the rhizosphere sediment samples from the N, SJ, SC and SR zones.**

| Samples | NH_4_^+^  mmol/kg | NO_2_^-^  μmol/kg | NO_3_^-^  μmol/kg | Cl^-^  mmol/kg | Br^-^  mmol/kg | SO_4_^2-^  mmol/kg | MD  μm | D50  μm | TON  % | TC  % | TOC  % |
| --- | --- | --- | --- | --- | --- | --- | --- | --- | --- | --- | --- |
| NRS1 | 0.739 | 2.765 | 0.093 | 156.385 | 0.203 | 5.978 | 71.313 | 27.665 | 0.096 | 0.833 | 0.579 |
| NRS2 | 0.944 | 0 | 0 | 181.409 | 0.308 | 6.757 | 92.089 | 50.916 | 0.083 | 0.715 | 0.720 |
| NRS3 | 0.630 | 1.835 | 4.236 | 147.412 | 0.198 | 6.053 | 69.479 | 27.991 | 0.058 | 0.553 | 0.488 |
| NRS4 | 1.163 | 3.121 | 0.450 | 164.535 | 0.166 | 6.438 | 61.484 | 21.063 | 0.082 | 0.663 | 0.505 |
| SJRS1 | 1.485 | 3.747 | 25.896 | 162.431 | 0.168 | 6.710 | 193.898 | 182.408 | 0.020 | 1.428 | 1.120 |
| SJRS2 | 0.928 | 2.774 | 29.012 | 364.874 | 0.489 | 15.786 | 201.670 | 190.816 | 0.030 | 2.312 | 2.142 |
| SJRS3 | 1.259 | 12.373 | 31.199 | 199.606 | 0.210 | 7.648 | 167.645 | 155.564 | 0.050 | 2.166 | 2.289 |
| SJRS4 | 1.071 | 3.159 | 4.698 | 164.924 | 0.215 | 6.283 | 175.072 | 153.157 | 0.033 | 3.095 | 2.861 |
| SCRS1 | 0.829 | 0 | 0 | 193.545 | 0.233 | 6.773 | 49.702 | 25.653 | 0.233 | 2.375 | 2.499 |
| SCRS2 | 0.919 | 0 | 0 | 205.091 | 0.247 | 5.870 | 113.899 | 34.587 | 0.290 | 3.020 | 2.667 |
| SCRS3 | 0.764 | 1.979 | 8.378 | 110.233 | 0.118 | 4.508 | 77.187 | 24.645 | 0.320 | 3.315 | 2.834 |
| SCRS4 | 0.907 | 1.957 | 4.114 | 224.695 | 0.289 | 7.614 | 40.694 | 22.420 | 0.346 | 3.429 | 2.636 |
| SRRS1 | 0.170 | 1.002 | 4.355 | 144.696 | 0.174 | 5.819 | 55.823 | 36.713 | 0.103 | 1.146 | 1.025 |
| SRRS2 | 0.424 | 1.733 | 13.624 | 158.777 | 0.204 | 6.250 | 35.106 | 23.308 | 0.105 | 1.186 | 1.121 |
| SRRS3 | 0.322 | 0.882 | 5.189 | 124.722 | 0.148 | 4.531 | 32.793 | 21.464 | 0.112 | 1.216 | 1.202 |
| SRRS4 | 0.178 | 1.044 | 0.742 | 184.161 | 0.221 | 7.166 | 51.741 | 33.053 | 0.104 | 1.157 | 1.127 |

**Table S5 The ASVs numbers, Chao 1, Shannon and Coverage indices**

| Samples | Bacterial community | | | | Eukaryotic community | | | | Fungal community | | | |
| --- | --- | --- | --- | --- | --- | --- | --- | --- | --- | --- | --- | --- |
|  | ASVs | Chao 1 | Shannon | Coverage | ASVs | Chao | Shannon | Coverage | ASVs | Chao | Shannon | Coverage |
| NSW1 | 455 | 454.06 | 3.98 | 99.94% | 592 | 616.12 | 3.49 | 99.70% | 369 | 365.18 | 3.75 | 99.92% |
| NSW2 | 465 | 466.53 | 4.05 | 99.94% | 606 | 608.01 | 3.67 | 99.78% | 300 | 300.50 | 3.73 | 99.96% |
| NSW3 | 557 | 557.31 | 4.33 | 99.92% | 742 | 749.15 | 3.64 | 99.71% | 260 | 267.50 | 4.58 | 99.96% |
| NSW4 | 661 | 426.50 | 4.06 | 99.95% | 433 | 431.35 | 3.50 | 99.98% | 394 | 394.80 | 4.62 | 99.93% |
| SJSW1 | 744 | 661.02 | 4.97 | 99.99% | 808 | 821.65 | 4.31 | 99.68% | 312 | 302.38 | 1.90 | 99.97% |
| SJSW2 | 823 | 744.35 | 5.51 | 99.96% | 284 | 284.00 | 2.73 | 100.00% | 581 | 574.74 | 3.80 | 99.90% |
| SJSW3 | 618 | 823.37 | 5.70 | 99.96% | 560 | 559.34 | 3.41 | 99.78% | 363 | 370.25 | 4.85 | 99.95% |
| SJSW4 | 426 | 621.09 | 4.99 | 99.92% | 171 | 178.33 | 2.11 | 99.93% | 587 | 575.89 | 3.78 | 99.88% |
| SCSW1 | 449 | 443.35 | 3.68 | 99.77% | 570 | 575.13 | 3.91 | 99.82% | 239 | 238.00 | 4.57 | 100.00% |
| SCSW2 | 753 | 758.74 | 5.05 | 99.84% | 676 | 672.10 | 4.59 | 99.88% | 191 | 192.50 | 3.70 | 99.98% |
| SCSW3 | 199 | 198.27 | 3.45 | 99.98% | 513 | 527.89 | 3.88 | 99.85% | 223 | 223.50 | 4.04 | 99.98% |
| SCSW4 | 300 | 300.00 | 3.77 | 100.00% | 768 | 759.42 | 5.23 | 99.93% | 459 | 454.97 | 4.46 | 99.92% |
| SRSW1 | 338 | 335.75 | 4.08 | 99.90% | 117 | 113.05 | 2.06 | 99.97% | 70 | 68.00 | 2.19 | 100.00% |
| SRSW2 | 329 | 330.17 | 4.15 | 99.90% | 99 | 102.56 | 1.71 | 99.94% | 102 | 102.33 | 2.17 | 99.99% |
| SRSW3 | 354 | 355.27 | 4.21 | 99.88% | 168 | 170.00 | 2.05 | 99.93% | 205 | 205.00 | 3.91 | 100.00% |
| SRSW4 | 340 | 342.77 | 4.05 | 99.89% | 80 | 80.75 | 1.69 | 99.97% | 231 | 232.50 | 3.79 | 99.98% |
| NRS1 | 1633 | 1644.99 | 6.60 | 99.30% | 1091 | 1080.24 | 4.14 | 99.54% | 611 | 618.53 | 4.66 | 99.87% |
| NRS2 | 1112 | 1113.14 | 6.37 | 99.83% | 434 | 430.10 | 1.52 | 99.78% | 507 | 519.50 | 3.92 | 99.86% |
| NRS3 | 1270 | 1271.66 | 6.46 | 99.71% | 982 | 985.26 | 3.74 | 99.61% | 435 | 437.00 | 3.56 | 99.93% |
| NRS4 | 1482 | 1491.46 | 6.60 | 99.53% | 963 | 974.78 | 3.84 | 99.53% | 461 | 462.48 | 2.52 | 99.86% |
| SJRS1 | 2163 | 2186.65 | 6.72 | 98.39% | 635 | 632.79 | 2.31 | 99.79% | 287 | 287.00 | 4.17 | 100.00% |
| SJRS2 | 1735 | 1729.00 | 6.72 | 99.29% | 981 | 981.37 | 3.77 | 99.68% | 513 | 513.33 | 4.92 | 99.98% |
| SJRS3 | 1964 | 1979.16 | 6.66 | 98.71% | 354 | 357.89 | 2.54 | 99.88% | 293 | 292.17 | 4.11 | 99.99% |
| SJRS4 | 1569 | 1595.97 | 6.54 | 99.22% | 891 | 902.24 | 3.54 | 99.51% | 468 | 467.05 | 4.84 | 99.99% |
| SCRS1 | 1177 | 1175.55 | 5.63 | 99.51% | 663 | 661.67 | 3.01 | 99.63% | 495 | 497.52 | 4.26 | 99.93% |
| SCRS2 | 1987 | 2014.65 | 6.63 | 98.85% | 832 | 832.17 | 4.60 | 99.98% | 665 | 660.88 | 4.83 | 99.87% |
| SCRS3 | 1416 | 1450.52 | 5.91 | 99.12% | 586 | 599.10 | 2.72 | 99.59% | 407 | 405.96 | 4.50 | 99.96% |
| SCRS4 | 1886 | 1932.29 | 6.54 | 98.77% | 659 | 667.32 | 3.45 | 99.83% | 369 | 370.64 | 4.22 | 99.97% |
| SRRS1 | 1310 | 1325.65 | 6.10 | 99.50% | 1147 | 1139.09 | 4.50 | 99.58% | 427 | 428.55 | 2.64 | 99.96% |
| SRRS2 | 1527 | 1538.23 | 6.38 | 99.31% | 367 | 371.45 | 2.71 | 99.91% | 273 | 273.27 | 1.37 | 99.98% |
| SRRS3 | 1577 | 1585.89 | 6.49 | 99.42% | 702 | 701.26 | 3.45 | 99.84% | 297 | 297.33 | 1.37 | 99.95% |
| SRRS4 | 1386 | 1382.33 | 6.23 | 99.54% | 1521 | 1524.30 | 5.05 | 99.50% | 397 | 396.75 | 2.26 | 99.94% |

**Table S6 The taxonomy assignment of metagenome-assembled genomes (MAGs) from all samples.**

| **Zones** | **Samples** | **MAGs** | **Classification** | **Taxonomy** |
| --- | --- | --- | --- | --- |
| NS | NSW1 | NSW1.bin.18 | Nanopelagicales bacterium | d__Bacteria;p__Actinomycetota;c__Actinomycetia;o__Nanopelagicales;f__S36-B12;g__UBA4592;s__UBA4592 sp002390705 |
|  |  | NSW1.bin.25 | *Yoonia* sp. | d__Bacteria;p__Pseudomonadota;c__Alphaproteobacteria;o__Rhodobacterales;f__Rhodobacteraceae;g__Yoonia;s__ |
|  |  | NSW1.bin.29 | *Wenyingzhuangia* sp. | d__Bacteria;p__Bacteroidota;c__Bacteroidia;o__Flavobacteriales;f__Flavobacteriaceae;g__Wenyingzhuangia;s__ |
|  |  | NSW1.bin.30 | *Nonlabens* sp. | d__Bacteria;p__Bacteroidota;c__Bacteroidia;o__Flavobacteriales;f__Flavobacteriaceae;g__Nonlabens;s__ |
|  |  | NSW1.bin.36 | Verrucomicrobiales bacterium | d__Bacteria;p__Verrucomicrobiota;c__Verrucomicrobiae;o__Verrucomicrobiales;f__DEV007;g__JAACDM01;s__JAACDM01 sp009936795 |
|  |  | NSW1.bin.42 | Flavobacteriales bacterium | d__Bacteria;p__Bacteroidota;c__Bacteroidia;o__Flavobacteriales;f__CAJJDF01;g__CAJJDF01;s__ |
|  |  | NSW1.bin.43 | *Alteriqipengyuania* sp | d__Bacteria;p__Pseudomonadota;c__Alphaproteobacteria;o__Sphingomonadales;f__Sphingomonadaceae;g__Alteriqipengyuania;s__Alteriqipengyuania sp002377695 |
|  |  | NSW1.bin.8 | *Planktotalea* arctica | d__Bacteria;p__Pseudomonadota;c__Alphaproteobacteria;o__Rhodobacterales;f__Rhodobacteraceae;g__Planktotalea;s__Planktotalea arctica |
|  | NSW2 | NSW2.bin.13 | *Celeribacter* marinus | d__Bacteria;p__Pseudomonadota;c__Alphaproteobacteria;o__Rhodobacterales;f__Rhodobacteraceae;g__Celeribacter;s__Celeribacter marinus |
|  |  | NSW2.bin.24 | *Planktotalea* arctica | d__Bacteria;p__Pseudomonadota;c__Alphaproteobacteria;o__Rhodobacterales;f__Rhodobacteraceae;g__Planktotalea;s__Planktotalea arctica |
|  |  | NSW2.bin.32 | Flavobacteriaceae bacterium | d__Bacteria;p__Bacteroidota;c__Bacteroidia;o__Flavobacteriales;f__Flavobacteriaceae;g__SCGC-AAA160-P02;s__SCGC-AAA160-P02 sp913060575 |
|  |  | NSW2.bin.35 | Flavobacteriales bacterium | d__Bacteria;p__Bacteroidota;c__Bacteroidia;o__Flavobacteriales;f__CAJJDF01;g__CAJJDF01;s__ |
|  |  | NSW2.bin.38 | *Octadecabacter* sp | d__Bacteria;p__Pseudomonadota;c__Alphaproteobacteria;o__Rhodobacterales;f__Rhodobacteraceae;g__Octadecabacter;s__Octadecabacter sp018500005 |
|  |  | NSW2.bin.42 | *Polaribacter* sp. | d__Bacteria;p__Bacteroidota;c__Bacteroidia;o__Flavobacteriales;f__Flavobacteriaceae;g__Polaribacter;s__Polaribacter sp018663625 |
|  |  | NSW2.bin.43 | *Alteriqipengyuania* sp | d__Bacteria;p__Pseudomonadota;c__Alphaproteobacteria;o__Sphingomonadales;f__Sphingomonadaceae;g__Alteriqipengyuania;s__Alteriqipengyuania sp002377695 |
|  | NSW3 | NSW3.bin.1 | *Yoonia* sp. | d__Bacteria;p__Pseudomonadota;c__Alphaproteobacteria;o__Rhodobacterales;f__Rhodobacteraceae;g__Yoonia;s__ |
|  |  | NSW3.bin.15 | Nanopelagicales bacterium | d__Bacteria;p__Actinomycetota;c__Actinomycetia;o__Nanopelagicales;f__S36-B12;g__UBA4592;s__UBA4592 sp002390705 |
|  |  | NSW3.bin.30 | *Nonlabens* sp. | d__Bacteria;p__Bacteroidota;c__Bacteroidia;o__Flavobacteriales;f__Flavobacteriaceae;g__Nonlabens;s__ |
|  |  | NSW3.bin.38 | *Planktotalea* *arctica* | d__Bacteria;p__Pseudomonadota;c__Alphaproteobacteria;o__Rhodobacterales;f__Rhodobacteraceae;g__Planktotalea;s__Planktotalea arctica |
|  |  | NSW3.bin.4 | Flavobacteriales bacterium | d__Bacteria;p__Bacteroidota;c__Bacteroidia;o__Flavobacteriales;f__CAJJDF01;g__CAJJDF01;s__ |
|  | NSW4 | NSW4.bin.12 | *Polaribacter* sp. | d__Bacteria;p__Bacteroidota;c__Bacteroidia;o__Flavobacteriales;f__Flavobacteriaceae;g__Polaribacter;s__ |
|  |  | NSW4.bin.16 | *Hellea* sp. | d__Bacteria;p__Pseudomonadota;c__Alphaproteobacteria;o__Caulobacterales;f__Maricaulaceae;g__Hellea;s__Hellea sp905477785 |
|  |  | NSW4.bin.26 | *Planktotalea* *arctica* | d__Bacteria;p__Pseudomonadota;c__Alphaproteobacteria;o__Rhodobacterales;f__Rhodobacteraceae;g__Planktotalea;s__Planktotalea arctica |
|  |  | NSW4.bin.29 | *Winogradskyella* sp. | d__Bacteria;p__Bacteroidota;c__Bacteroidia;o__Flavobacteriales;f__Flavobacteriaceae;g__Winogradskyella;s__ |
|  |  | NSW4.bin.30 | Flavobacteriales bacterium | d__Bacteria;p__Bacteroidota;c__Bacteroidia;o__Flavobacteriales;f__CAJJDF01;g__CAJJDF01;s__ |
|  |  | NSW4.bin.4 | Flavobacteriaceae bacterium | d__Bacteria;p__Bacteroidota;c__Bacteroidia;o__Flavobacteriales;f__Flavobacteriaceae;g__SCGC-AAA160-P02;s__SCGC-AAA160-P02 sp913060575 |
|  |  | NSW4.bin.45 | *Nonlabens* sp. | d__Bacteria;p__Bacteroidota;c__Bacteroidia;o__Flavobacteriales;f__Flavobacteriaceae;g__Nonlabens;s__ |
|  |  | NSW4.bin.6 | *Octadecabacter* sp | d__Bacteria;p__Pseudomonadota;c__Alphaproteobacteria;o__Rhodobacterales;f__Rhodobacteraceae;g__Octadecabacter;s__Octadecabacter sp018500005 |
|  |  | NSW4.bin.7 | *Celeribacter* *marinus* | d__Bacteria;p__Pseudomonadota;c__Alphaproteobacteria;o__Rhodobacterales;f__Rhodobacteraceae;g__Celeribacter;s__Celeribacter marinus |
|  | NRS2 | NRS2.bin.1 | Acetivibrionales bacterium | d__Bacteria;p__Bacillota_A;c__Clostridia;o__Acetivibrionales_B;f__;g__;s__ |
| SC | SCSW1 | SCSW1.bin.11 | Flavobacteriales bacterium | d__Bacteria;p__Bacteroidota;c__Bacteroidia;o__Flavobacteriales;f__CAJJDF01;g__CAJJDF01;s__CAJJDF01 sp024639505 |
|  |  | SCSW1.bin.15 | *Lentimonas* sp. | d__Bacteria;p__Verrucomicrobiota;c__Verrucomicrobiae;o__Opitutales;f__DSM-45221;g__Lentimonas;s__ |
|  |  | SCSW1.bin.16 | Akkermansiaceae bacterium | d__Bacteria;p__Verrucomicrobiota;c__Verrucomicrobiae;o__Verrucomicrobiales;f__Akkermansiaceae;g__SW10;s__ |
|  |  | SCSW1.bin.22 | Akkermansiaceae bacterium | d__Bacteria;p__Verrucomicrobiota;c__Verrucomicrobiae;o__Verrucomicrobiales;f__Akkermansiaceae;g__SW10;s__ |
|  |  | SCSW1.bin.28 | *Polaribacter* sp. | d__Bacteria;p__Bacteroidota;c__Bacteroidia;o__Flavobacteriales;f__Flavobacteriaceae;g__Polaribacter;s__Polaribacter sp018663625 |
|  |  | SCSW1.bin.29 | *Pacificibacter* sp. | d__Bacteria;p__Pseudomonadota;c__Alphaproteobacteria;o__Rhodobacterales;f__Rhodobacteraceae;g__Pacificibacter;s__ |
|  |  | SCSW1.bin.3 | *Arcticimaribacter* sp. | d__Bacteria;p__Bacteroidota;c__Bacteroidia;o__Flavobacteriales;f__Flavobacteriaceae;g__Arcticimaribacter;s__ |
|  |  | SCSW1.bin.31 | *Qipengyuania* flava | d__Bacteria;p__Pseudomonadota;c__Alphaproteobacteria;o__Sphingomonadales;f__Sphingomonadaceae;g__Qipengyuania;s__Qipengyuania flava |
|  |  | SCSW1.bin.33 | *Glaciecola* sp. | d__Bacteria;p__Pseudomonadota;c__Gammaproteobacteria;o__Enterobacterales_A;f__Alteromonadaceae;g__Glaciecola;s__Glaciecola sp000155775 |
|  |  | SCSW1.bin.37 | *Polaribacter* sp. | d__Bacteria;p__Bacteroidota;c__Bacteroidia;o__Flavobacteriales;f__Flavobacteriaceae;g__Polaribacter;s__ |
|  |  | SCSW1.bin.43 | *Hyphomonas* sp. | d__Bacteria;p__Pseudomonadota;c__Alphaproteobacteria;o__Caulobacterales;f__Hyphomonadaceae;g__Hyphomonas;s__Hyphomonas sp000682775 |
|  |  | SCSW1.bin.51 | *Polaribacter* sp. | d__Bacteria;p__Bacteroidota;c__Bacteroidia;o__Flavobacteriales;f__Flavobacteriaceae;g__Polaribacter;s__ |
|  |  | SCSW1.bin.9 | *Abyssibacter* sp. | d__Bacteria;p__Pseudomonadota;c__Gammaproteobacteria;o__Nevskiales;f__OUC007;g__Abyssibacter;s__ |
|  | SCSW2 | SCSW2.bin.10 | Akkermansiaceae bacterium | d__Bacteria;p__Verrucomicrobiota;c__Verrucomicrobiae;o__Verrucomicrobiales;f__Akkermansiaceae;g__SW10;s__ |
|  |  | SCSW2.bin.21 | Flavobacteriales bacterium | d__Bacteria;p__Bacteroidota;c__Bacteroidia;o__Flavobacteriales;f__CAJJDF01;g__CAJJDF01;s__CAJJDF01 sp024639505 |
|  |  | SCSW2.bin.22 | *Wenyingzhuangia* sp. | d__Bacteria;p__Bacteroidota;c__Bacteroidia;o__Flavobacteriales;f__Flavobacteriaceae;g__Wenyingzhuangia;s__ |
|  |  | SCSW2.bin.23 | *Pacificibacter* sp. | d__Bacteria;p__Pseudomonadota;c__Alphaproteobacteria;o__Rhodobacterales;f__Rhodobacteraceae;g__Pacificibacter;s__ |
|  |  | SCSW2.bin.24 | *Lentimonas* sp. | d__Bacteria;p__Verrucomicrobiota;c__Verrucomicrobiae;o__Opitutales;f__DSM-45221;g__Lentimonas;s__ |
|  |  | SCSW2.bin.3 | *Glaciecola* sp. | d__Bacteria;p__Pseudomonadota;c__Gammaproteobacteria;o__Enterobacterales_A;f__Alteromonadaceae;g__Glaciecola;s__Glaciecola sp000155775 |
|  |  | SCSW2.bin.30 | *Polaribacter* sp. | d__Bacteria;p__Bacteroidota;c__Bacteroidia;o__Flavobacteriales;f__Flavobacteriaceae;g__Polaribacter;s__ |
|  |  | SCSW2.bin.33 | Akkermansiaceae bacterium | d__Bacteria;p__Verrucomicrobiota;c__Verrucomicrobiae;o__Verrucomicrobiales;f__Akkermansiaceae;g__SW10;s__ |
|  |  | SCSW2.bin.42 | *Arcticimaribacter* sp. | d__Bacteria;p__Bacteroidota;c__Bacteroidia;o__Flavobacteriales;f__Flavobacteriaceae;g__Arcticimaribacter;s__ |
|  | SCSW3 | SCSW3.bin.12 | *Glaciecola* sp. | d__Bacteria;p__Pseudomonadota;c__Gammaproteobacteria;o__Enterobacterales_A;f__Alteromonadaceae;g__Glaciecola;s__Glaciecola sp000155775 |
|  |  | SCSW3.bin.14 | *Arcticimaribacter* sp. | d__Bacteria;p__Bacteroidota;c__Bacteroidia;o__Flavobacteriales;f__Flavobacteriaceae;g__Arcticimaribacter;s__ |
|  |  | SCSW3.bin.23 | *Wenyingzhuangia* sp. | d__Bacteria;p__Bacteroidota;c__Bacteroidia;o__Flavobacteriales;f__Flavobacteriaceae;g__Wenyingzhuangia;s__ |
|  |  | SCSW3.bin.26 | *Lentimonas* sp. | d__Bacteria;p__Verrucomicrobiota;c__Verrucomicrobiae;o__Opitutales;f__DSM-45221;g__Lentimonas;s__ |
|  |  | SCSW3.bin.3 | *Glaciecola* sp. | d__Bacteria;p__Pseudomonadota;c__Gammaproteobacteria;o__Enterobacterales_A;f__Alteromonadaceae;g__Glaciecola;s__ |
|  |  | SCSW3.bin.36 | Akkermansiaceae bacterium | d__Bacteria;p__Verrucomicrobiota;c__Verrucomicrobiae;o__Verrucomicrobiales;f__Akkermansiaceae;g__SW10;s__ |
|  |  | SCSW3.bin.49 | *Polaribacter* sp. | d__Bacteria;p__Bacteroidota;c__Bacteroidia;o__Flavobacteriales;f__Flavobacteriaceae;g__Polaribacter;s__ |
|  |  | SCSW3.bin27 | Flavobacteriales bacterium | d__Bacteria;p__Bacteroidota;c__Bacteroidia;o__Flavobacteriales;f__CAJJDF01;g__CAJJDF01;s__CAJJDF01 sp024639505 |
|  | SCSW4 | SCSW4.bin.16 | Flavobacteriales bacterium | d__Bacteria;p__Bacteroidota;c__Bacteroidia;o__Flavobacteriales;f__CAJJDF01;g__CAJJDF01;s__CAJJDF01 sp024639505 |
|  |  | SCSW4.bin.19 | Akkermansiaceae bacterium | d__Bacteria;p__Verrucomicrobiota;c__Verrucomicrobiae;o__Verrucomicrobiales;f__Akkermansiaceae;g__SW10;s__ |
|  |  | SCSW4.bin.29 | *Lentimonas* sp. | d__Bacteria;p__Verrucomicrobiota;c__Verrucomicrobiae;o__Opitutales;f__DSM-45221;g__Lentimonas;s__ |
|  |  | SCSW4.bin.33 | *Wenyingzhuangia* sp. | d__Bacteria;p__Bacteroidota;c__Bacteroidia;o__Flavobacteriales;f__Flavobacteriaceae;g__Wenyingzhuangia;s__ |
|  |  | SCSW4.bin.39 | *Arcticimaribacter* sp. | d__Bacteria;p__Bacteroidota;c__Bacteroidia;o__Flavobacteriales;f__Flavobacteriaceae;g__Arcticimaribacter;s__ |
|  |  | SCSW4.bin.4 | *Glaciecola* sp. | d__Bacteria;p__Pseudomonadota;c__Gammaproteobacteria;o__Enterobacterales_A;f__Alteromonadaceae;g__Glaciecola;s__Glaciecola sp000155775 |
| SJ | SJRS3 | SJRS3.bin.13 | Desulfobacterales bacterium | d__Bacteria;p__Desulfobacterota;c__Desulfobacteria;o__Desulfobacterales;f__SG8-13;g__;s__ |
|  |  | SJRS3.bin.5 | *Thiogranum* sp. | d__Bacteria;p__Pseudomonadota;c__Gammaproteobacteria;o__DSM-19610;f__DSM-19610;g__Thiogranum;s__ |
|  |  | SJRS3.bin.9 | Flavobacteriaceae bacterium | d__Bacteria;p__Bacteroidota;c__Bacteroidia;o__Flavobacteriales;f__Flavobacteriaceae;g__JAHEJJ01;s__ |
|  | SJRS4 | SJRS4.bin.22 | Desulfobacterales bacterium | d__Bacteria;p__Desulfobacterota;c__Desulfobacteria;o__Desulfobacterales;f__SG8-13;g__;s__ |
|  |  | SJRS4.bin.3 | *Thiogranum* sp. | d__Bacteria;p__Pseudomonadota;c__Gammaproteobacteria;o__DSM-19610;f__DSM-19610;g__Thiogranum;s__ |
|  |  | SJRS4.bin9 | Flavobacteriaceae bacterium | d__Bacteria;p__Bacteroidota;c__Bacteroidia;o__Flavobacteriales;f__Flavobacteriaceae;g__JAHEJJ01;s__ |
|  | SJSW1 | SJSW1.bin.1 | Akkermansiaceae bacterium | d__Bacteria;p__Verrucomicrobiota;c__Verrucomicrobiae;o__Verrucomicrobiales;f__Akkermansiaceae;g__SW10;s__ |
|  |  | SJSW1.bin.21 | Flavobacteriales bacterium | d__Bacteria;p__Bacteroidota;c__Bacteroidia;o__Flavobacteriales;f__CAJJDF01;g__CAJJDF01;s__CAJJDF01 sp018609055 |
|  |  | SJSW1.bin.39 | *Polaribacter* sp. | d__Bacteria;p__Bacteroidota;c__Bacteroidia;o__Flavobacteriales;f__Flavobacteriaceae;g__Polaribacter;s__Polaribacter sp024640565 |
|  |  | SJSW1.bin.9 | *Lentimonas* sp. | d__Bacteria;p__Verrucomicrobiota;c__Verrucomicrobiae;o__Opitutales;f__DSM-45221;g__Lentimonas;s__ |
|  | SJSW2 | SJSW2.bin.21 | *Lentimonas* sp. | d__Bacteria;p__Verrucomicrobiota;c__Verrucomicrobiae;o__Opitutales;f__DSM-45221;g__Lentimonas;s__ |
|  |  | SJSW2.bin.4 | Akkermansiaceae bacterium | d__Bacteria;p__Verrucomicrobiota;c__Verrucomicrobiae;o__Verrucomicrobiales;f__Akkermansiaceae;g__SW10;s__ |
|  |  | SJSW2.bin.46 | Bacteroidia bacterium | d__Bacteria;p__Bacteroidota;c__Bacteroidia;o__NS11-12g;f__UBA9320;g__UBA9320;s__UBA9320 sp018700895 |
|  | SJSW3 | SJSW3.bin.19 | *Lentimonas* sp. | d__Bacteria;p__Verrucomicrobiota;c__Verrucomicrobiae;o__Opitutales;f__DSM-45221;g__Lentimonas;s__ |
|  |  | SJSW3.bin.44 | Akkermansiaceae bacterium | d__Bacteria;p__Verrucomicrobiota;c__Verrucomicrobiae;o__Verrucomicrobiales;f__Akkermansiaceae;g__SW10;s__ |
|  |  | SJSW3.bin.48 | Alphaproteobacteria bacterium | d__Bacteria;p__Pseudomonadota;c__Alphaproteobacteria;o__SPJN01;f__SPJN01;g__SPJN01;s__SPJN01 sp006844605 |
|  |  | SJSW3.bin.69 | *Polaribacter* sp. | d__Bacteria;p__Bacteroidota;c__Bacteroidia;o__Flavobacteriales;f__Flavobacteriaceae;g__Polaribacter;s__ |
|  |  | SJSW3.bin.7 | Oceanococcaceae bacterium | d__Bacteria;p__Pseudomonadota;c__Gammaproteobacteria;o__Nevskiales;f__Oceanococcaceae;g__MS8;s__ |
|  | SJSW4 | SJSW4.bin.16 | Akkermansiaceae bacterium | d__Bacteria;p__Verrucomicrobiota;c__Verrucomicrobiae;o__Verrucomicrobiales;f__Akkermansiaceae;g__SW10;s__ |
|  |  | SJSW4.bin.21 | *Polaribacter* sp. | d__Bacteria;p__Bacteroidota;c__Bacteroidia;o__Flavobacteriales;f__Flavobacteriaceae;g__Polaribacter;s__Polaribacter sp024640565 |
|  |  | SJSW4.bin.24 | *Lentimonas* sp. | d__Bacteria;p__Verrucomicrobiota;c__Verrucomicrobiae;o__Opitutales;f__DSM-45221;g__Lentimonas;s__ |
|  |  | SJSW4.bin.48 | Bacteroidia bacterium | d__Bacteria;p__Bacteroidota;c__Bacteroidia;o__NS11-12g;f__UBA9320;g__UBA9320;s__UBA9320 sp018700895 |
| SR | SRRS1 | SRRS1.bin.17 | Desulfobacterales bacterium | d__Bacteria;p__Desulfobacterota;c__Desulfobacteria;o__Desulfobacterales;f__SG8-13;g__;s__ |
|  | SRRS2 | SRRS2.bin.17 | Desulfobacterales bacterium | d__Bacteria;p__Desulfobacterota;c__Desulfobacteria;o__Desulfobacterales;f__SG8-13;g__;s__ |
|  |  | SRRS2.bin.4 | Arenicellales bacterium | d__Bacteria;p__Pseudomonadota;c__Gammaproteobacteria;o__Arenicellales;f__BMS3Bbin11;g__BMS3Bbin11;s__ |
|  |  | SRRS2.bin.6 | *Sulfurovum* sp. | d__Bacteria;p__Campylobacterota;c__Campylobacteria;o__Campylobacterales;f__Sulfurovaceae;g__Sulfurovum;s__ |
|  | SRRS3 | SRRS3.bin.4 | Desulfuromonadales bacterium | d__Bacteria;p__Desulfobacterota_F;c__Desulfuromonadia;o__Desulfuromonadales;f__BM103;g__JAFDBW01;s__ |
|  | SRRS4 | SRRS4.bin.17 | Desulfobacterales bacterium | d__Bacteria;p__Desulfobacterota;c__Desulfobacteria;o__Desulfobacterales;f__SG8-13;g__;s__ |
|  |  | SRRS4.bin.4 | Desulfuromonadales bacterium | d__Bacteria;p__Desulfobacterota_F;c__Desulfuromonadia;o__Desulfuromonadales;f__BM103;g__JAFDBW01;s__ |
|  | SRSW2 | SRSW2.bin.2 | *Alteriqipengyuania* sp. | d__Bacteria;p__Pseudomonadota;c__Alphaproteobacteria;o__Sphingomonadales;f__Sphingomonadaceae;g__Alteriqipengyuania;s__Alteriqipengyuania sp002377695 |
|  |  | SRSW2.bin.29 | *Pseudophaeobacter* *gallaeciensis* | d__Bacteria;p__Pseudomonadota;c__Alphaproteobacteria;o__Rhodobacterales;f__Rhodobacteraceae;g__Pseudophaeobacter;s__Pseudophaeobacter gallaeciensis |
|  |  | SRSW2.bin.4 | *Nocardioides* *marinisabuli* | d__Bacteria;p__Actinomycetota;c__Actinomycetia;o__Propionibacteriales;f__Nocardioidaceae;g__Nocardioides;s__Nocardioides marinisabuli |
|  |  | SRSW2.bin.40 | Verrucomicrobiales bacterium | d__Bacteria;p__Verrucomicrobiota;c__Verrucomicrobiae;o__Verrucomicrobiales;f__DEV007;g__Arctic95D-9;s__Arctic95D-9 sp022424805 |
|  |  | SRSW2.bin.5 | *Marivita* sp. | d__Bacteria;p__Pseudomonadota;c__Alphaproteobacteria;o__Rhodobacterales;f__Rhodobacteraceae;g__Marivita;s__ |
|  |  | SRSW2.bin.9 | Arcobacteraceae bacterium | d__Bacteria;p__Campylobacterota;c__Campylobacteria;o__Campylobacterales;f__Arcobacteraceae;g__JALRJW01;s__ |
|  | SRSW3 | SRSW3.bin.13 | Flavobacteriaceae bacterium | d__Bacteria;p__Bacteroidota;c__Bacteroidia;o__Flavobacteriales;f__Flavobacteriaceae;g__MAG-120531;s__MAG-120531 sp022239325 |
|  |  | SRSW3.bin.22 | *Polycyclovorans* sp. | d__Bacteria;p__Pseudomonadota;c__Gammaproteobacteria;o__Nevskiales;f__Nevskiaceae;g__Polycyclovorans;s__Polycyclovorans sp002706265 |
|  |  | SRSW3.bin.34 | *Ketobacter* sp | d__Bacteria;p__Pseudomonadota;c__Gammaproteobacteria;o__Pseudomonadales;f__Ketobacteraceae;g__Ketobacter;s__Ketobacter sp002471665 |
|  |  | SRSW3.bin.5 | *Nocardioides* sp. | d__Bacteria;p__Actinomycetota;c__Actinomycetia;o__Propionibacteriales;f__Nocardioidaceae;g__Nocardioides;s__Nocardioides sp002698575 |
|  |  | SRSW3.bin.52 | Rhodobacteraceae bacterium | d__Bacteria;p__Pseudomonadota;c__Alphaproteobacteria;o__Rhodobacterales;f__Rhodobacteraceae;g__JALQTR01;s__ |
|  |  | SRSW3.bin.65 | *Marivita* sp. | d__Bacteria;p__Pseudomonadota;c__Alphaproteobacteria;o__Rhodobacterales;f__Rhodobacteraceae;g__Marivita;s__ |
|  | SRSW4 | SRSW4.bin.56 | *Marivita* sp. | d__Bacteria;p__Pseudomonadota;c__Alphaproteobacteria;o__Rhodobacterales;f__Rhodobacteraceae;g__Marivita;s__ |
|  |  | SRSW4.bin.65 | Akkermansiaceae bacterium | d__Bacteria;p__Verrucomicrobiota;c__Verrucomicrobiae;o__Verrucomicrobiales;f__Akkermansiaceae;g__SW10;s__ |

**References**

1. Lin Z, Ye W, Zu X, Xie H, Li H, Li Y et al. Integrative metabolic and microbial profiling on patients with Spleen-yang-deficiency syndrome. Sci Rep. 2018;8(1):6619.

2. Stoeck T, Bass D, Nebel M, Christen R, Jones MD, Breiner HW et al. Multiple marker parallel tag environmental DNA sequencing reveals a highly complex eukaryotic community in marine anoxic water. Mol Ecol. 2010;19 Suppl 1(21-31.

3. Adams RI, Miletto M, Taylor JW, Bruns TD. Dispersal in microbes: fungi in indoor air are dominated by outdoor air and show dispersal limitation at short distances. ISME J. 2013;7(7):1262-1273.
